# Astrocyte-derived SerpinA3N promotes neuroinflammation and epileptic seizures by activating the NF-κB signaling pathway in mice with temporal lobe epilepsy

**DOI:** 10.1186/s12974-023-02840-8

**Published:** 2023-07-08

**Authors:** Chong Liu, Xue-Min Zhao, Qiao Wang, Ting-Ting Du, Mo-Xuan Zhang, Hui-Zhi Wang, Ren-Peng Li, Kun Liang, Yuan Gao, Si-Yu Zhou, Tao Xue, Jian-Guo Zhang, Chun-Lei Han, Lin Shi, Liang-Wen Zhang, Fan-Gang Meng

**Affiliations:** 1 Beijing Neurosurgical Institute, Capital Medical University, Beijing, 100070 China; 2 Beijing Key Laboratory of Neurostimulation, Beijing, 100070 China; 3 Beijing Tiantan Hospital, Capital Medical University, No. 119 South Fourth Ring West Road, Fengtai District, Beijing, 100070 China; 4 Department of Neurosurgery, Shandong Provincial Hospital Affiliated to Shandong First Medical University, Jinan, 250021 Shandong China; 5 Chinese Institute for Brain Research, Beijing, 102206 China

**Keywords:** Astrocytes, SerpinA3N, Neuroinflammation, Epileptic seizures, Temporal lobe epilepsy, Multi-omics analysis

## Abstract

Impaired activation and regulation of the extinction of inflammatory cells and molecules in injured neuronal tissues are key factors in the development of epilepsy. SerpinA3N is mainly associated with the acute phase response and inflammatory response. In our current study, transcriptomics analysis, proteomics analysis, and Western blotting showed that the expression level of Serpin clade A member 3N (SerpinA3N) is significantly increased in the hippocampus of mice with kainic acid (KA)-induced temporal lobe epilepsy, and this molecule is mainly expressed in astrocytes. Notably, in vivo studies using gain- and loss-of-function approaches revealed that SerpinA3N in astrocytes promoted the release of proinflammatory factors and aggravated seizures. Mechanistically, RNA sequencing and Western blotting showed that SerpinA3N promoted KA-induced neuroinflammation by activating the NF-κB signaling pathway. In addition, co-immunoprecipitation revealed that SerpinA3N interacts with ryanodine receptor type 2 (RYR2) and promotes RYR2 phosphorylation. Overall, our study reveals a novel SerpinA3N-mediated mechanism in seizure-induced neuroinflammation and provides a new target for developing neuroinflammation-based strategies to reduce seizure-induced brain injury.

**Supplementary Information:**

The online version contains supplementary material available at 10.1186/s12974-023-02840-8.

## Introduction

Temporal lobe epilepsy (TLE) is the most common type of chronic epilepsy in adults, affecting up to 70 million people worldwide [1–3]. The molecular mechanism of intractable epilepsy remains unclear. Many different neurobiological processes are considered potential targets for anti-epileptic therapy [4]. These processes include neurodegeneration, neurogenesis, neuroinflammation, changes in neuronal ion channels, neurotransmitter release or uptake, intracellular signaling cascades, metabolic changes, and blood‒brain barrier integrity [3, 5–7].

Many of these processes are thought to be driven by epigenomic changes induced by epileptogenic insult [3]. Although in some hereditary or sporadic cases, we have found genes associated with epilepsy, these findings do not fully reveal the pathogenesis of epilepsy [8–10]. Identification of the causative gene of epilepsy may be challenging because the same epileptic phenotype may be associated with multiple genes [11]. High-throughput microarrays and RNA sequencing (RNA-seq) allow an analysis of gene expression in a comprehensive, unbiased, and genome-wide manner without presuming candidate genes to identify new genes and molecular pathways associated with the disease. Proteins are direct drug targets and are the main mediators of biological activities. Proteomics provides a global view of protein changes at the functional or network level. In the past two decades, this method has been widely used in animal models and patients with epilepsy and other diseases, and many differentially expressed genes have been found [12–17]. However, concerns about the representativeness and repeatability of single microarray or RNA-seq analysis [18] and proteomics analysis [19, 20] have also been raised, and the heterogeneity of different experimental designs, animal models, sample sites, acquisition times, and sample sizes may confuse the interpretation of the results.

Serpin clade A member 3 (SerpinA3) is a member of the serine protease inhibitor (Serpin) superfamily and is primarily used as a protease inhibitor for maintaining cellular homeostasis [21]. This molecule is a stromal cell acute phase glycoprotein that appears to be the only nuclear-bound secretory serpin [22]. For the human gene, the mouse serine protease inhibitor A3 family exists in 14 gene clusters located on chromosome 12F1 (named serine protease inhibitors A3a-n) [21], which has 70% homology with human genes [23]. Therefore, it is considered the functional ortholog of human SerpinA3 in the brain [24]. Recently, SerpinA3N/SerpinA3 has been found to be significantly upregulated in neurological diseases, such as traumatic brain injury, Alzheimer's disease, ischemic stroke, hippocampal stab injury, glioma, hypothalamus inflammation, and plays an important role in the development of the disease [25–30]. Moreover, SerpinA3N is mainly associated with the acute phase response and inflammatory response [31]. SerpinA3N reduces neuronal apoptosis and neuroinflammation by activating the Akt–mTOR pathway after stroke in the acute phase [27]. Pre-treatment of lymphocytes with SerpinA3N prevented GrB-mediated axonal and neuronal injury, as well as demyelination in multiple sclerosis [32]. Sinensetin, a natural flavonoid with anti-inflammatory and antioxidant properties, attenuates IL-1β-induced cartilage damage and ameliorates osteoarthritis by regulating SerpinA3 [33]. However, it was also reported SerpinA3N knockout could attenuate airway hyperreactivity, mitigate inflammatory responses and reduce collagen deposition in lung tissues of neonatal mice with asthma [34]. In addition, another important role was found to be a new potential marker of reactive astrocyte proliferation and hypothalamic inflammation during CNS injury [31]. It was reported that SerpinA3N not only plays a key role in TMT-induced neuroinflammation and neurotoxicity, but also acts as a key target to exert anti-inflammatory effects [35]. However, the function and regulatory mechanism of SerpinA3N in epilepsy have not been clarified.

Here, we performed transcriptomics and proteomics analysis of the same time segment, the same epilepsy model, and the same part of the sample and combined analysis to explore the mRNA and protein expression profiles in the hippocampus of mice with TLE to identify the key molecules in the pathogenesis of epilepsy. We found that SerpinA3N was highly expressed in the hippocampus of mice with TLE and promoted hippocampal neuroinflammation, aggravated seizures, and neuronal loss. Our findings will help to elucidate the molecular mechanisms of KA-induced neuroinflammation and provide new options for its treatment.

## Materials and methods

### Animal and human samples

C57BL/6 mice, 8 weeks old and weighing 21–23 g, were purchased from Vital River Laboratory Animal Technology Co., Ltd. (Beijing, China). The mice were housed in cages with a temperature of 20 ± 2 °C, relative humidity of 60% ± 10%, and a 12-h light/dark cycle. All efforts were made to minimize the number of animals used and animal suffering in this study. Cortical specimens were obtained from patients with mesial TLE and traumatic brain injury who underwent surgical treatment in Beijing Tiantan Hospital. Control cortical tissue was taken from autopsy patients without a history of epilepsy or other neurological diseases. Written informed consent was obtained from each patient for the use of brain tissues for research purposes. The present research was approved by the Ethics Committee of Beijing Neurosurgical Institute, Capital Medical University, Beijing, China (Process No. 201902029 and KY 2021-067-02).

### Epilepsy models and video-EEG monitoring

Based on our previous research experience [36], 0.3 µl of kainic acid (KA) (1 µg/L, Sigma-Aldrich, St. Louis, MO, USA) was stereotactically injected into the right amygdala (0.94 mm posterior to the bregma, 2.75 mm lateral to the midline, and 4.75 mm ventral to the bregma) by a stereotaxic apparatus (David Kopf Instruments, Tujunga, CA, USA). The animals in the sham group were injected with the same volume of saline, but all other steps were the same. The development of seizures was evaluated by Racine’s scale assessment [37]. To maximize the success rate of the model and reduce the mortality rate, we injected diazepam (6 mg/kg, ip), when the mice experienced Racine stage 4/5 seizures and status epilepticus (SE) for 60 min. The sham-operated controls received the same volume of an intra-amygdala saline injection. Three weeks after KA or saline injection, three screws with EEG recording wires were implanted into the right frontal bone and bilateral suboccipital bone and fixed with dental cement. One week later, 7 d of uninterrupted video-EEG monitoring was performed. The mice were decapitated immediately or perfused transcardially with PBS and 4% paraformaldehyde after anesthesia with an appropriate amount of isoflurane. We stored the brain tissues in a − 80 °C freezer or fixed them with PBS and 4% paraformaldehyde for future analysis.

### Drug administration

Triptolide (PG490) (CAT#S3604, Selleck Chemicals, Texas, USA) is a triepoxide diterpene compound that is an immunosuppressant extracted from the Chinese herbal medicine *Tripterygium wilfordii*. This molecule is an NF-κB inhibitor that inhibits NF-κB transcriptional activity, disrupts the p65/CBP interaction, and reduces p65 protein levels. Two weeks after empty virus, SerpinA3N overexpression (OE), and SerpinA3N knockdown AVV injection, KA was injected into the right amygdala to induce epilepsy. The sham-operated control animals received saline injections. PG490 (300 µg/kg) or 0.5% dimethyl sulfoxide (DMSO) was injected intraperitoneally half an hour before KA injection, once a day from day 2 to day 7.

### Transcriptome and quantitative proteomics analysis

Transcriptome analysis was performed by SHBIO Biotechnology Corporation (Shanghai, China) and BGI-Tech (Shenzhen, China). Quantitative proteomics analysis was performed by Jingjie PTM Bio Lab Co., Ltd. (Hangzhou, China). Detailed procedures are described in the supplementary materials and methods.

### Construction and stereotactic injection of SerpinA3N overexpression and silencing adeno-associated virus (AAV)

A vector harboring SerpinA3N (OE-SerpinA3N) or short hairpin RNA targeting SerpinA3N (shRNA) was constructed by VectorBuilder (Guangzhou, China) and Hanbio Tech Co., Ltd., (Shanghai, China), respectively. The expression of SerpinA3N was driven by the GFAP promoter. The serotype AAV9 was selected for investigation in this study because in previous studies they have been characterized to exhibit the greatest propensity astrocytes in various CNS regions including the spinal cord by various constitutive promoters or glial-specific promoters [38–42]. Detailed procedures are described in the supplementary materials and methods. A total of 0.5 μl of AAV was infused into the right dorsal hippocampus (2.1 mm posterior to the bregma, 1.5 mm lateral to the midline, and 1.75 mm ventral to the bregma) through a syringe pump (KDS Legato 100, KD Scientific, USA) at a speed of 0.1 μl/min. The needle was held in place for 5 min and then slowly withdrawn.

### Quantitative polymerase chain reaction (qPCR) analysis

Total RNA was extracted and purified using a QIAGEN RNeasy Mini Kit (Cat# 74106, QIAGEN, GmBH, Germany) following the manufacturer's instructions. The total RNA was then subjected to quality inspection using a Nanodrop ND-2000 spectrophotometer (Nanodrop Technologies). Reverse transcription was performed using a first-strand cDNA synthesis kit according to the manufacturer’s instructions (Cat#KR116-02, Tiangen, Beijing, China). qPCR was performed with RealStar Green Fast Mixture (Cat#A304-10, GenStar BioSolutions Co., Ltd., Beijing, China) in a 20 µl system containing 10 µl of RealStar Green Fast Mixture, 0.25 µl of each primer (20 µM), 1 µl of cDNA sample, and 8.5 µl of ddH_2_O using the QuantStudio™ 5 Real-Time PCR System (Thermo Fisher Scientific) under the following conditions: 95 °C for 2 min followed by 40 cycles of 15 s at 95 °C and 30 s at 60 °C. The primer sequences are listed in Additional file 5: Table S4. Each sample was analyzed in triplicate. The PCR products were confirmed by melting curve analysis. Relative expression levels were normalized to Gapdh using the 2^−ΔΔCt^ method.

### Western blot analysis and co-immunoprecipitation (Co-IP)

Samples were harvested and homogenized. A standard procedure was carried out as described previously [43]. Briefly, total protein was extracted from hippocampal or cortical tissue, and 50–70 µg of total protein was separated by MOPS-PAGE, transferred to PVDF membranes, blocked with 5% skim milk at room temperature for 2 h, and incubated in a 4 °C refrigerator after adding the corresponding antibody overnight. Co-IP was conducted according to the manufacturer’s instructions (abs955, Absin Bioscience, Inc., China). Fresh hippocampal tissue was lysed with 300 µl of lysis buffer for 20 min on ice, and the cell lysates (approximately 280 μl) were incubated with 5 μl of protein agarose A/G beads at 4 °C for 45 min. The supernatant was collected and incubated with 5.0 μg corresponding antibody or IgG overnight on a rotating plate; 5 μl of agarose A/G beads was then added to each sample and incubated at 4 °C for 1 h. Finally, the precipitate was washed three times with washing buffer and subjected to Western blot analysis. Detailed procedures are described in the supplementary materials and methods. Protein band density was scanned with an Epson V330 Photo scanner (Seiko Epson Co.) or a ChemiDoc imaging system (Bio-Rad) and quantified using ImageJ software. Uncropped Western blots are provided in the Supplementary Material.

### Immunofluorescence (IF) analysis

A standard procedure was carried out as described previously [36]. Briefly, coronal sections (8 μm) were prepared at the level of the dorsal hippocampus (1.90–2.50 mm posterior to the bregma). Paraffin sections were dried, washed, permeabilized, blocked in 5% goat serum, and incubated overnight at 4 °C with antibodies against goat anti-SerpinA3N (RD; AF4709), mouse anti-NeuN (Millipore; MAB377), rabbit anti-Olig2 (Abcam; ab109186), rabbit anti-GFAP (Abcam; ab4648), rabbit anti-IBA1 (Huabio; ET1705-78), rabbit anti-phospho-RYR2 (Ser2808) (Biorbyt; orb1093816), and mouse anti-RYR2 (Thermo Fisher Scientific, MA3-916), followed by an Alexa Fluor 680-, 594-, or 488-conjugated secondary antibody (Thermo Fisher Scientific, A-21057, A-11058, A-11001, A-11008) for 1 h at room temperature. Finally, the cells were incubated with mounting medium (with DAPI) (Cat#S2110, Solarbio, Beijing, China) at room temperature for 15 min. Histological images were captured using a Pannoramic MIDI scanner (3D Histech, Hungary). The experiment was repeated in triplicate. ImageJ software was used to manually count positive cells in the same size field of view.

### Nissl staining

Coronal sections (25 μm) from the level of the dorsal hippocampus (1.80–3.0 mm posterior to bregma) were analyzed. Tissue sections were stained with cresyl violet (Cat#C0117, Beyotime Institute of Biotechnology, Jiangsu, China) according to the manufacturer's protocol. Brain sections were stained with 1% toluidine blue for 10 min at 37 °C. The slides were then rinsed in distilled water 2 times for 2 min each time, dehydrated in 95% ethanol 2 times for 2 min each time, clarified in xylene for 5 min, and coverslipped with neutral balsam. The total cell numbers in the CA3, CA1, and dentate hilus areas of the hippocampus were counted from three nonoverlapping 400 × fields of each section (Olympus BX41, Olympus Optical Co., Ltd., Japan) using a computer-assisted image analysis system (Leica Qwin Analysis software V2.8). All procedures were conducted by a pathologist who was blinded to the grouping to avoid any bias in cell counting. Five coronary sections were collected, and a total of three sections per animal were used for quantification. ImageJ software was used to manually count positive cells in the same size field of view.

### Microarray datasets of epilepsy and bioinformatics analysis

The microarray datasets (GSE122228, GSE73878, GSE71058, and GSE127871) were obtained by searching the Gene Expression Omnibus (GEO) database. Detailed sample information is described in Additional file 6: Table S5. GEO2R was used to screen for differential expression between the hippocampus of epileptic and control mice. A fold change cutoff ≥ 1.5 with a p value cutoff < 0.05 was considered to be statistically significant.

### Statistical analysis

All data are presented as the mean ± standard error of the mean (SEM). Statistical analysis was performed using GraphPad Prism 8.0 Software. The effects of treatments were analyzed using Student’s *t* test when comparing two groups and by one-way ANOVA or two-way ANOVA followed by Dunnett’s multiple comparisons test when comparing more than two groups. Significance was accepted at *p* < 0.05.

## Results

### Identification of differential expression of proteins and mRNAs in the hippocampus of mice with TLE by transcriptomics and proteomics

**Fig. 1:**
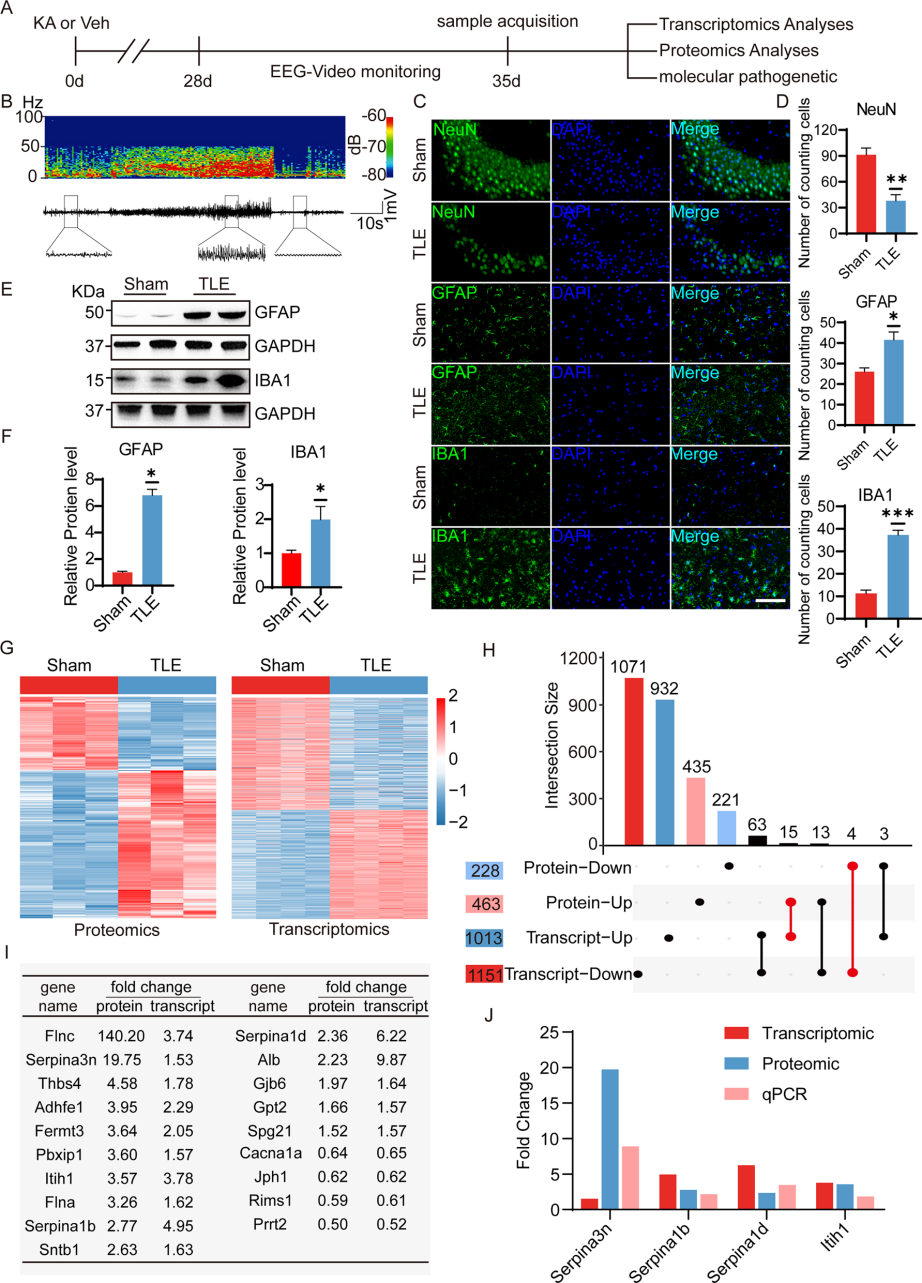
Differential expression of proteins and mRNAs in the hippocampus of mice with TLE. **A** Experimental timeline of the transcriptomics and proteomics. **B** An EEG time–frequency spectrogram of a typical mouse SRS of chronic epilepsy. **C**, **D** Immunostaining and quantitative analysis for NeuN, GFAP, and Iba1 cells number in the CA3 region of the hippocampus from the KA-induced epileptic mice at 35 d after surgery (scale bar = 100 µm; *n* = 4). Normalized to the sham level. **E**, **F** Western blotting and densitometric quantitative analysis of GFAP and Iba1 in the hippocampus from the KA-induced epileptic mice at 35 d after surgery (*n* = 4). Normalized to the sham level. GAPDH served as the internal control. **G** Heatmap showing the clustering of the differential expression of proteins and mRNAs (top 1000) in the hippocampus of the mice with TLE. The color scale shows proteins or mRNAs that are upregulated (red) or downregulated (blue) relative to the mean expression of all samples. **H** UpSet diagram showing the intersection between the differential expression of proteins and mRNAs in the hippocampus of the mice with TLE. **I** Fold changes of the 19 differentially expressed genes in transcriptomics and proteomics. **J** The fold changes in the expression levels of 4 mRNAs tested by transcriptomics, proteomics and qPCR (*n* = 3–4). All data are shown as the mean ± SEM. *p < 0.05; **p < 0.01. SRS: spontaneous recurrent seizures; KA: kainic acid; TLE: temporal lobe epilepsy

Following the experimental procedure described in Fig. 1A, intra-amygdala kainic acid injection successfully induced a mouse model of temporal lobe epilepsy. Mice with spontaneous recurrent seizures (SRS) were selected by video-EEG monitoring for subsequent experiments. The EEG time–frequency spectrogram of a typical mouse SRS is shown in Fig. 1B. Western blotting and immunofluorescence staining were used to detect molecular pathological changes in the hippocampus at 35 d after SE. Compared with those in the sham group, neurons in the CA3, CA1, and DG regions were significantly lost, and the number of GFAP- and IBA1-positive cells was significantly increased (Fig. 1C, D and Additional file 1: Fig. S1A, B). Similarly, the protein levels of GFAP and IBA1 in the hippocampus of the mice with TLE were significantly increased (Fig. 1E, F).

To identify the relationship between transcripts and proteins related to temporal lobe epilepsy, we performed transcriptomic and proteomic analysis of the hippocampus in the mice with SRS at 35 d after KA injection and detected a total of 46,868 transcript sequences and 6085 proteins (Additional file 2: Tables S1, Additional file 3: Table S2). We obtained 2164 differentially expressed genes and 691 proteins (FC ≥ 1.5 or FC ≤ 0.667, *p* value < 0.05). As shown in the heatmap with clustering of proteins and transcript sequences (top 1000) in the hippocampus, there were obvious differences between the sham mice and the mice with TLE (Fig. 1G). The combined analysis of the two groups obtained 19 differentially expressed molecules with the same expression trend at the protein level and gene level (Fig. 1H, I). qPCR was used to analyze the mRNA levels of randomly selected Serpina3n, Serpina1b, Serpina1d, and Itih1 in the KA 35 d mice. The mean levels of Serpina3n mRNA were increased most significantly (Additional file 1: Fig. S2A). Based on transcriptomics, proteomics and qPCR mean fold change (Fig. 1J), we speculate that SerpinA3N plays an important role in TLE.

### SerpinA3N was highly expressed in the hippocampus of mice with TLE and mainly expressed in hippocampal astrocytes

**Fig. 2:**
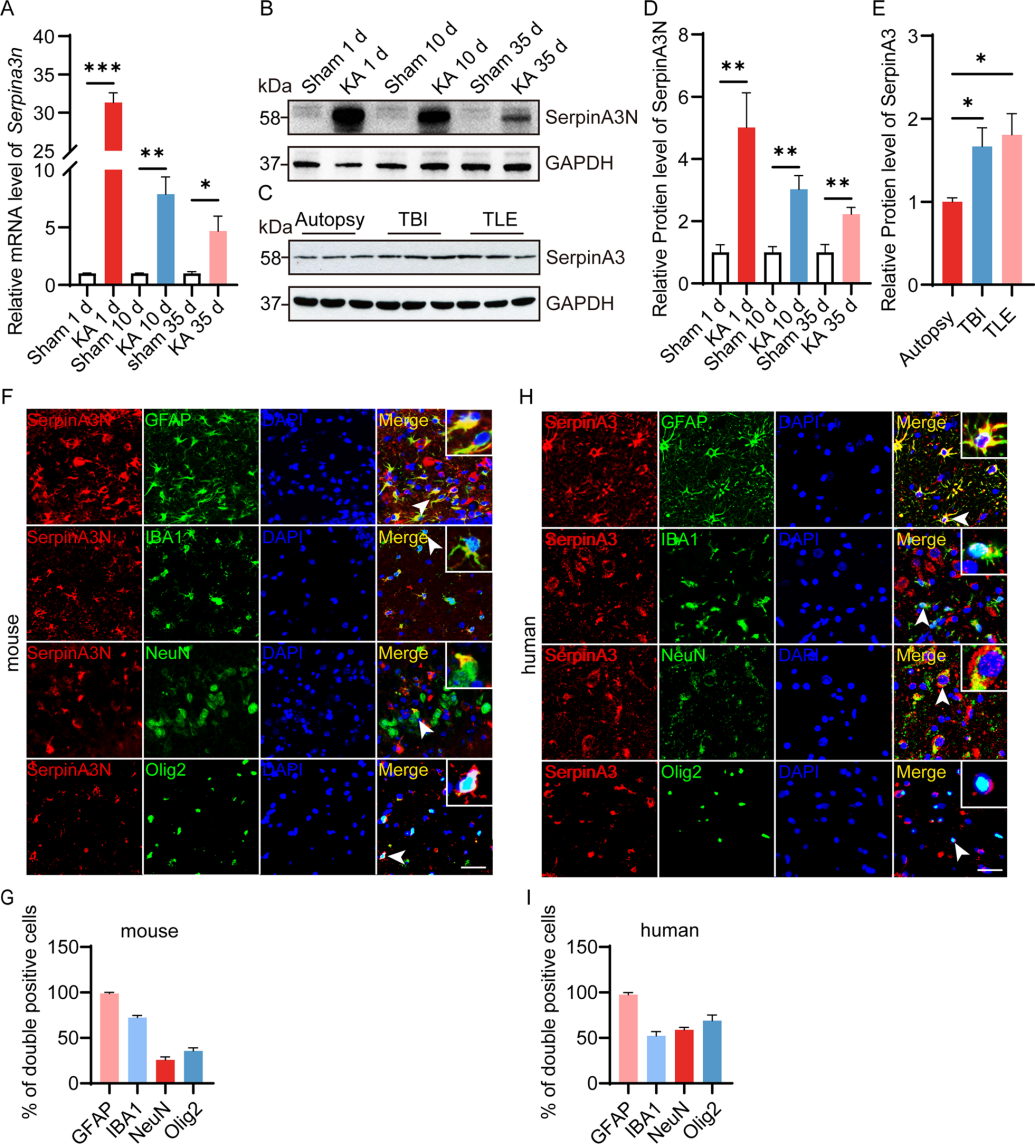
SerpinA3N was highly expressed in TLE mice and mainly expressed in hippocampal astrocytes. **A** The expression of SerpinA3N was determined by qPCR in the hippocampus of epileptic mice at 1, 10, and 35 d after KA injection (*n* = 4). Normalized to the sham level. Gapdh served as the internal control. **B**, **D** Western blotting and densitometric quantitative analysis of SerpinA3N in the hippocampus of epileptic mice at 1, 10, and 35 d after KA injection (*n* = 3). Normalized to the sham level. GAPDH served as the internal control. **C**, **E** Western blotting and densitometric quantitative analysis of SerpinA3 in the temporal cortex of autopsy patients and TBI and TLE patients (*n* = 3). Normalized to autopsy patients’ level. GAPDH served as the internal control. **F**, **H** Immunostaining of SerpinA3N with the cell markers GFAP, Iba1, NeuN, and Olig2 in the hippocampal CA3 region of epileptic mice at 1 day after KA injection and the hippocampus of TLE patients. Scale bar = 50 μm. **G**, **I** Percentage of cells that are double-positive for SerpinA3N and GFAP/Iba1/NeuN or Olig2 among the total cells that are positive for GFAP/Iba1/NeuN or Olig2. All data are shown as the mean ± SEM. *p < 0.05, **p < 0.01, ***p < 0.001. KA: kainic acid; TBI: traumatic brain injury; TLE: temporal lobe epilepsy

To fully explore the SerpinA3N expression pattern in different periods of TLE, we used an intra-amygdala injection KA-induced mouse model for qPCR and Western blotting analyses of SerpinA3N expression. As shown in Fig. 2A, B, D, the RNA and protein expression levels of SerpinA3N was upregulated in the SE-experienced mice at 1, 10, and 35 d after KA injection. In addition, we obtained transcriptomic expression data of the hippocampus of epileptic models from the GEO database (GSE73878, GSE122228). The results showed that the expression level of Serpina3n increased in SE-experienced mice 7, 14, 28, and 60 d after KA injection by GEO2R (Additional file 1: Fig. S2B). SerpinA3N shares 70% homology with SerpinA3 in human [23], Western blotting showed that the expression of SerpinA3 in the hippocampus of patients with TLE and the cortex of patients with traumatic brain injury was higher than that in autopsy patients (Fig. 2C, E). By analyzing the differential expression of GSE71058 and GSE127871, we found that the expression level of SerpinA3 was higher in TLE patients with hippocampal sclerosis (HS) than in those without HS (Additional file 1: Fig. S2C). A significant positive correlation was found between the number of seizures per month and the expression level of SerpinA3 (Additional file 1: Fig. S2D, E), which seems to indicate that SerpinA3 can be used as a biomarker for the severity of seizures.

To clarify the cell localization and spatial distribution pattern of SerpinA3N in the hippocampus, we simultaneously stained SerpinA3N/SerpinA3 in the four main neural cell types of the central nervous system (astrocytes, neurons, microglia and oligodendrocytes) by immunofluorescence staining in the hippocampus at 35 d after KA injection and in TLE patients. Immunofluorescence analysis showed that SerpinA3N was expressed mainly in hippocampal astrocytes, although we could also detect a minor expression of the protein in microglia, oligodendrocytes and neurons in mice with TLE (Fig. 2F), as well as in the hippocampus of TLE patients (Fig. 2G). More interestingly, SerpinA3N/SerpinA3 was present in almost all astrocytes in the hippocampus of the mice with TLE and the patients (Fig. 2H, I). Compared with the sham group, the number of SerpinA3N and GFAP double-positive cells increased significantly in hippocampal tissues at 1, 10, and 35 d after KA injection (Additional file 1: Fig. S3A, B). The above results fully show that the expression of SerpinA3N/SerpinA3 is upregulated in mice and patients with TLE and is expressed in almost all hippocampal astrocytes.

### Astrocyte-derived SerpinA3N promotes the inflammatory response in the hippocampus of mice with TLE

As shown in previous research reports, SerpinA3N is mainly related to the acute phase response and tissue inflammatory response [31]. Astrocytes (especially reactive astrocytes) can produce cytokines such as IL-1β, IL-6, TNF-α [44]. Our study shows that SerpinA3N is most widely distributed in hippocampal astrocytes. In this study, we constructed a SerpinA3N OE and short hairpin RNA (shRNA) AAV delivery system in astrocytes. Additional file 1: Fig. S4 shows the schematic representation of a modified AAV vector for the delivery of SerpinA3N overexpression and knockdown. The in vivo biological functions of SerpinA3N were investigated by SerpinA3N overexpression and knockdown in astrocytes using an AAV9 delivery system. The results of fluorescence observation on brain slices showed that injected AAV virus (AAV9-mCherry) had transduced the hippocampus and expression was seen only in the astrocytes (Additional file 1: Fig. S5A, B). qPCR and western blotting results showed that the application of OE-SerpinA3N and shRNA-SerpinA3N significantly increased and decreased the expression of SerpinA3N in the hippocampus, respectively (Additional file 1: Fig. S5C–E).

**Fig. 3:**
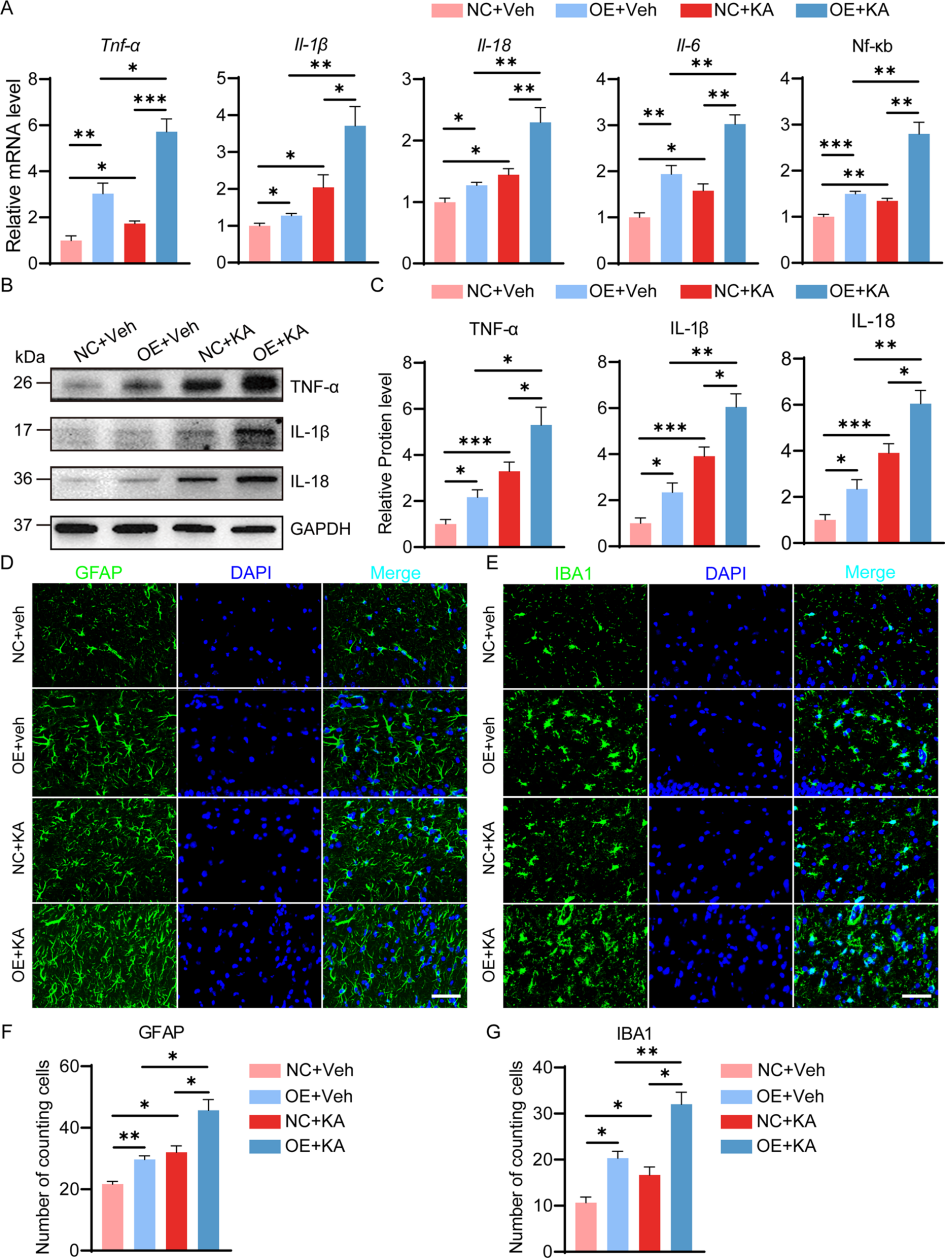
SerpinA3N overexpression aggravates KA-induced neuroinflammation and increases the number of hippocampal astrocytes and microglia in vivo. **A** The mRNA levels of TNF-α, Il-1β, Il-18, Il-6, and NF-κB in the hippocampus of SerpinA3N-overexpressing mice at 35 d post-SE, as determined by qPCR (*n* = 4). Normalized to the NC + veh level. GAPDH served as the internal control. **B**, **C** Western blotting and densitometric quantitative analysis of TNF-α, IL-1β and IL-18 in the hippocampal CA3 area of SerpinA3N-overexpressing mice at 35 d post-SE (*n* = 4). Normalized to the NC + veh level. GAPDH served as the internal control. **D**, **E** Representative fluorescence micrographs of GFAP and IBA1 expression in the hippocampus CA3 of SerpinA3N-overexpressing mice with or without KA treatment for 35 days. Scale bar = 50 μm. **F**, **G** The panels show the cell counts of cells positive for GFAP and IBA1 (*n* = 4). All data are shown as the mean ± SEM. *p < 0.05, **p < 0.01, ***p < 0.001. SE: status epilepticus; NC: empty AAV vectors; Veh: vehicle; KA: kainic acid; OE: AAV vectors containing overexpression serpina3n

**Fig. 4:**
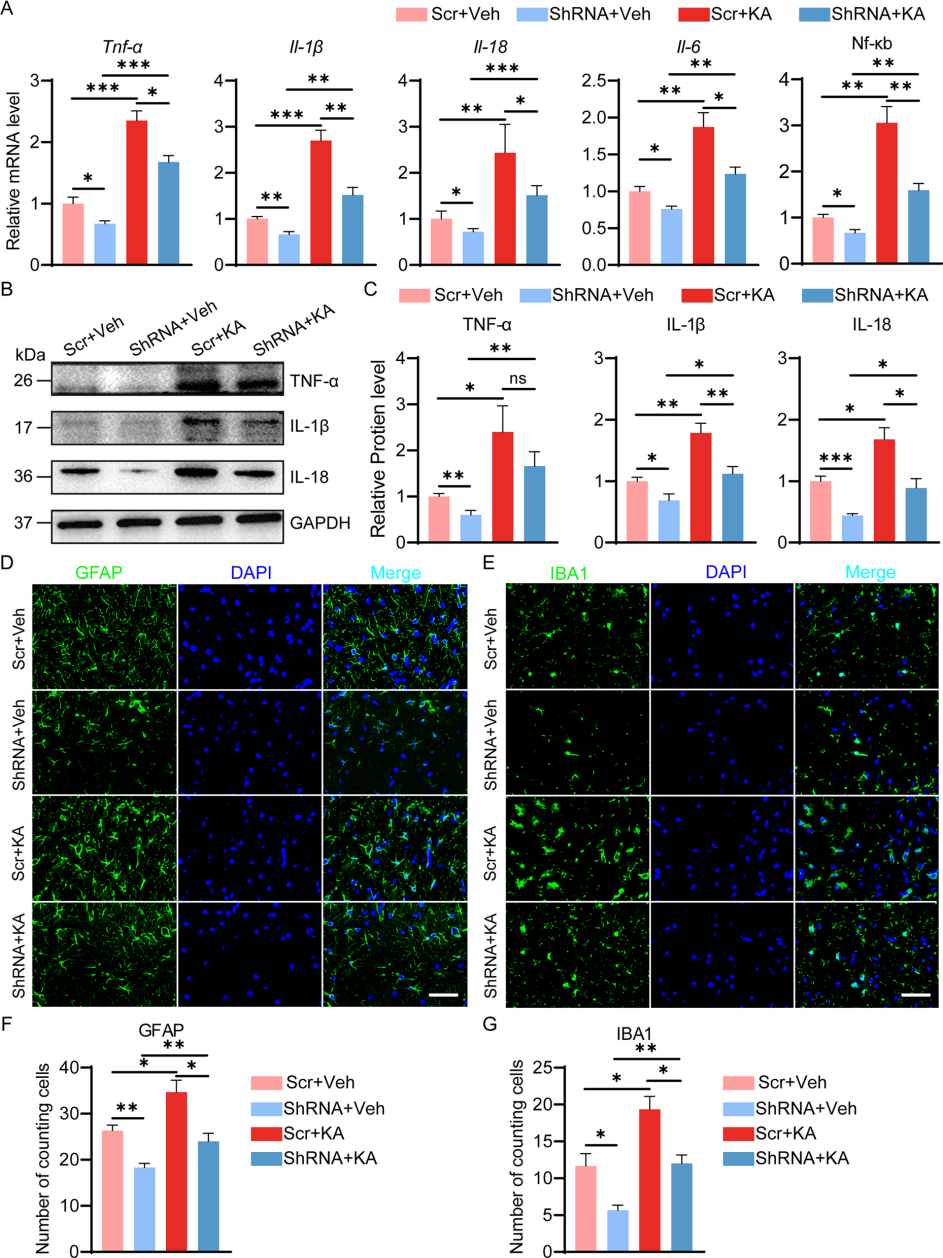
SerpinA3N knockdown alleviates KA-induced neuroinflammation and decreases the number of hippocampal astrocytes and microglia in vivo. **A** The mRNA levels of Tnf-α, Il-1β, Il-18, Il-6, and Nf-κb in the hippocampus of SerpinA3N knockdown mice at 35 d post-SE, as determined by qPCR (*n* = 4). Normalized to the Scr + veh level. GAPDH served as the internal control. **B**, **C** Western blotting and densitometric quantitative analysis of TNF-a, IL-1β and IL-18 in the hippocampus of SerpinA3N knockdown mice at 35 d post-SE (*n* = 4). Normalized to the Scr + veh level. GAPDH served as the internal control. **D**, **E** Representative fluorescence micrographs of GFAP and IBA1 expression in the hippocampal CA3 area of SerpinA3N knockdown mice with or without KA treatment for 35 days. Scale bar = 50 μm. **F**, **G** The panels show the cell counts of cells positive for GFAP and IBA1 (*n* = 3). All data are shown as the mean ± SEM. *p < 0.05, **p < 0.01, ***p < 0.001. SE: status epilepticus; Scr: scrambled AAV vectors; veh: vehicle; KA: kainic acid; ShRNA: AAV vectors containing short hairpin RNA targeting SerpinA3N

To clarify the effect of SerpinA3N on the inflammatory response of the hippocampus, we detected the expression levels of inflammation-related molecules in mice after SerpinA3N overexpression and knockdown. qPCR results showed that the expression levels of Tnf-α, Il-1β, Il-18, Il-6, and Nf-κb were increased in the hippocampus of epileptic mice and SerpinA3N-overexpressing mice, and SerpinA3N knockdown reversed these changes (Figs. 3A, 4A), indicating that KA and SerpinA3N can induce the upregulation of inflammatory factors alone. Furthermore, SerpinA3N overexpression exacerbated the upregulation of these mRNAs in the mice at 35 d after KA injection, SerpinA3N knockdown reversed these changes (Figs. 3A, 4A). In addition, Western blotting was used to further confirm the results (Figs. 3B, 4B).

More, immunofluorescence staining of GFAP and IBA1 showed that the distribution and number of astrocytes and microglia were affected in the hippocampus by KA-induced SE or SerpinA3N. Compared with the sham treatment, KA-induced SE or SerpinA3N overexpression alone increased the number of astrocytes or microglia in the hippocampus CA3 (Figs. 3D–G; 4D-G). KA-induced increases in a number of astrocytes and microglia were partially inhibited by SerpinA3N knockdown (Fig. 3D–G). More astrocytes and microglia were observed in the hippocampus CA3 of the mice overexpressing SerpinA3N at day 35 after SE (Fig. 4D–G). These results showed that SerpinA3N could enhance the inflammatory response and increase the number of astrocytes and microglia in epileptic mice.

### SerpinA3N promotes seizures and neuronal loss in mice with TLE

**Fig. 5:**
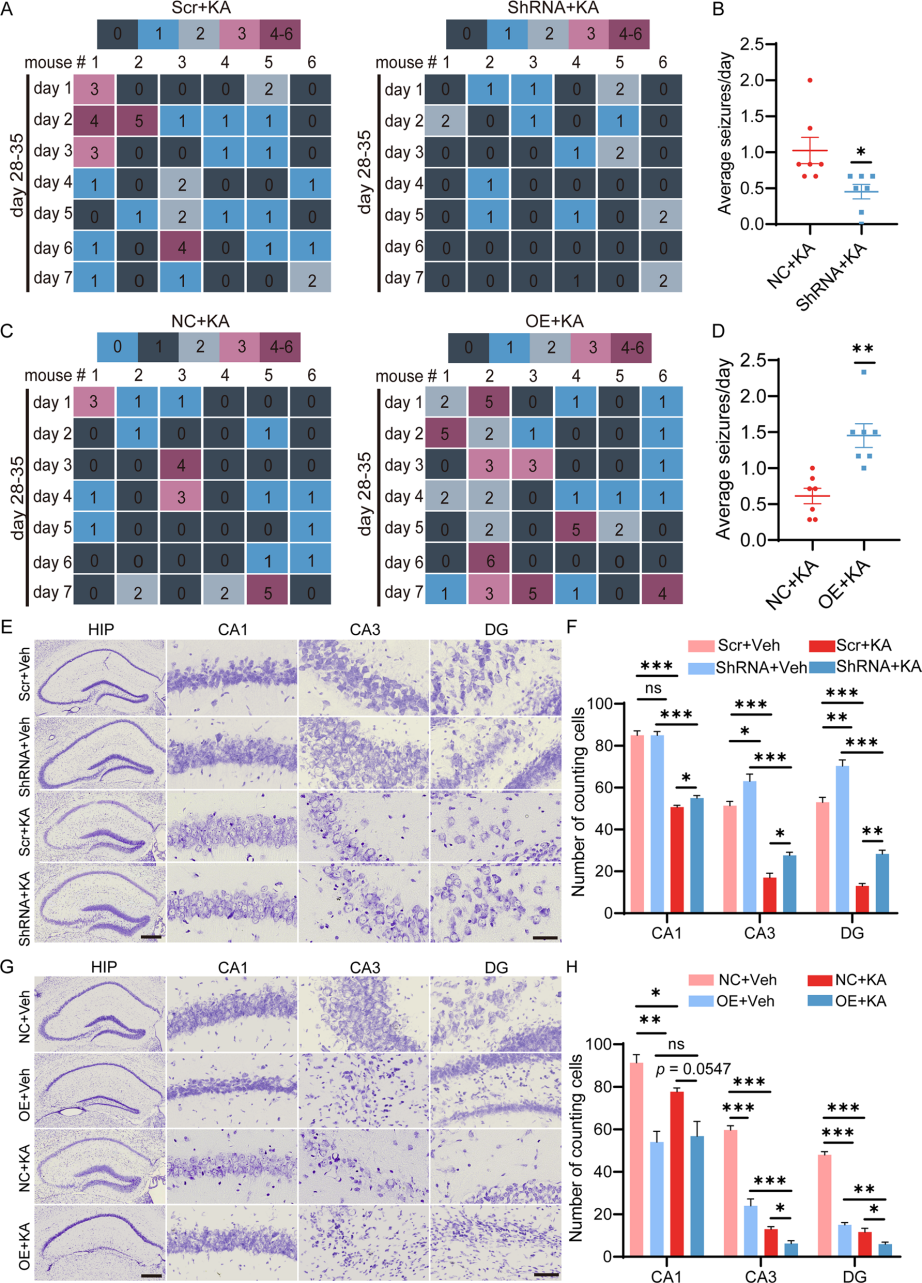
SerpinA3N aggravates seizures and KA-induced hippocampal neuronal loss in vivo. **A**, **B** SRS number and the mean daily frequency of the epileptic mice with or without SerpinA3N knockdown from 28 to 35 d post-SE by video monitoring (*n* = 6). **C**, **D** SRS number and the mean daily frequency of the epileptic rats with or without overexpression of SerpinA3N from 28 to 35 d post-SE by video monitoring (*n* = 6). **E**, **G** Representative Nissl staining photomicrographs of the ipsilateral hippocampus in response to AAV vectors and/or KA injection from SerpinA3N-overexpressing and SerpinA3N knockdown mice with or without KA treatment for 35 days (*n* = 3–4). **F**, **H** The panels show the counts of cells in the CA1, CA3, and DG regions of the hippocampus ipsilateral to the AAV vectors and/or KA injection side (*n* = 3–4). All data are shown as the mean ± SEM. ns, not significant. *p < 0.05, **p < 0.01, ***p < 0.001. SRS: spontaneous recurrent seizures; SE: status epilepticus; AAV: adeno-associated virus; KA: kainic acid; HIP: hippocampus

Video-EEG monitoring were performed to explore whether SerpinA3N influenced the occurrence and development of epilepsy. Additional file 1: Fig. S6A shows typical behavioral changes within 1 h after KA injection. Overexpression or knockdown of SerpinA3N did not accelerate or alleviate the progression of seizures after KA injection (Additional file 1: Fig. S6B). The frequency and duration of SRSs from 28 to 35 d after KA injection in mice with TLE with or without overexpression and knockdown of SerpinA3N were analyzed. Typical EEG time–frequency spectrograms of SRS after KA injection for 28–35 d are shown in Additional file 1: Fig. S6C. Figure 5A, C shows the number of seizures per day in mice with TLE. Knockdown of SerpinA3N partially reversed the frequency of KA-induced seizures (Fig. 5B). In contrast, overexpression of SerpinA3N increased the frequency of seizures (Fig. 5D). In addition, knockdown of SerpinA3N shortened the average duration of epilepsy, but there was no significant difference (Additional file 1: Fig. S6D). However, overexpression of SerpinA3N resulted in a significant prolongation of the average duration of epilepsy (Additional file 1: Fig. S6D).

Nissl staining was used to observe the effects of SerpinA3N on hippocampal neuronal loss. Mice at 35 d after SE and SerpinA3N overexpression alone without KA treatment resulted in neuronal loss in the hippocampus (Fig. 5E–H). This finding may be due to the damage caused to hippocampal neurons by inflammation induced by SerpinA3N overexpression. Knockdown of SerpinA3N partially inhibited KA-induced neuronal loss in the CA1, CA3 and DG (Fig. 5E, F). In contrast, overexpression of SerpinA3N pretreatment aggravated the loss of hippocampal neurons, and the neuronal loss of CA3 and DG was more obvious (Fig. 5G, H). In conclusion, behavioral analysis and Nissl staining showed that SerpinA3N promoted SRS severity and aggravated hippocampal neuron loss in mice with TLE.

### SerpinA3N activates the NF-κB signaling pathway in mice with TLE

**Fig. 6:**
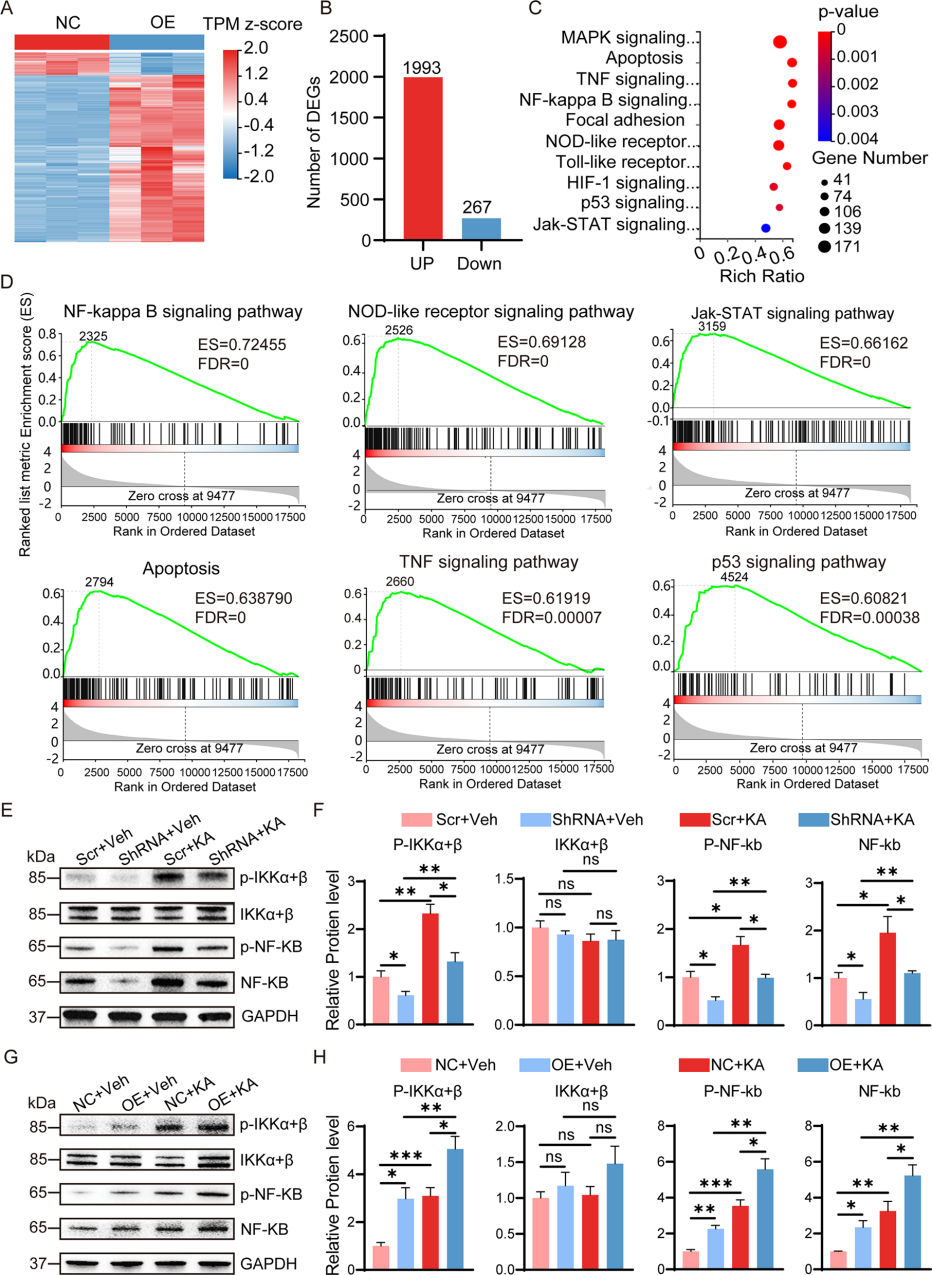
SerpinA3N activates the NF-κB signaling pathway in mice with TLE. **A** Heatmap with clustering of differentially expressed genes (top 1000) in the hippocampus of mice with SerpinA3N overexpression at 14 d after AAV vector injection. The color scale shows genes that are upregulated (red) or downregulated (blue) relative to the mean expression of all samples (*n* = 3). **B** The numbers of differentially expressed genes. **C** KEGG pathway enrichment analysis (ranked by p value). Dot size represents the number of genes in each KEGG pathway. The gene ratio (x-axis) is the percentage of significant genes over the total genes in a given pathway. **D** GSEA of differentially expressed genes. In each enrichment plot, the green curve corresponds to the ES curve, which is the running sum of the weighted ES. The FDR estimates the statistical significance of a single gene set's enrichment score. **E**–**H** Western blotting and densitometric quantitative analysis of NF-κB (p65), p-NF-κB (p65), p-IKKα + β, and IKKα + β in response to AAV vectors and/or KA injection from SerpinA3N-overexpressing and SerpinA3N knockdown mice with or without KA treatment for 35 days. GAPDH served as the internal control (*n* = 4). All data are shown as the mean ± SEM. Ns represents no significance. *p < 0.05, **p < 0.01, ***p < 0.001. AAV: adeno-associated virus; KEGG: Kyoto Encyclopedia of Genes and Genomes; GSEA: gene set enrichment analysis; ES: enrichment score; KA: kainic acid

To clarify the mechanism by which SerpinA3N promotes the hippocampal inflammatory response in mice with TLE, we performed RNA-seq analysis of hippocampal tissue after 14 d of overexpression of SerpinA3N. The heatmap analysis showed that there were significant differences between the two groups after overexpression of SerpinA3N, and the differentially expressed genes were mainly upregulated genes (Fig. 6A). Compared with NC group, 2260 differentially expressed genes (FC ≥ 2 or FC ≤ 0.5, *p* < 0.05) were identified, with 1993 upregulated and 267 downregulated genes (Fig. 6B) (Additional file 4: Table S3). KEGG pathway analysis revealed that inflammatory pathways were significantly enriched, including the NF-κB signaling pathway, TNF signaling pathway, and NOD-like receptor signaling pathway (Fig. 6C). Similarly, GSEA also found that gene sets associated with inflammation were significantly enriched (Fig. 6D). Among them, the enrichment score of the NF-κB signaling pathway was the highest. We speculate that SerpinA3N may promote inflammatory responses in mice with TLE by activating the NF-κB signaling pathway.

First, Western blot analysis showed that overexpression of SerpinA3N without KA treatment and KA-induced SE at 35 d increased the protein levels of NF-κB (p65), p-NF-κB (p65), and p-IKKα + β in the hippocampus, and SerpinA3N knockdown reversed these changes (Fig. 6E–H), indicating that KA and SerpinA3N can induce the upregulation of inflammatory factors alone. Furthermore, SerpinA3N overexpression exacerbated the alterations in the expression of these proteins in the mice after SE, while SerpinA3N knockdown prevented the SE-induced increase in NF-κB (p65), p-NF-κB (p65), and p-IKKα + β expression in the mice at 35 d after SE (Fig. 6E–H). There was no significant change in the protein expression level of IKKα + β in all groups.

Triptolide (PG490) is widely used as a specific inhibitor of the NF-κB signaling pathway. We injected PG490 intraperitoneally within 7 d after KA-induced epilepsy and evaluated the effect of PG490 on neuroinflammation by Western blotting. Post-treatment with PG490 successfully inhibited the protein levels of NF-κB (p65), p-NF-κB (p65), upstream p-IKKα + β, and downstream TNF-α, IL-1β, and IL-18 (Additional file 1: Fig. S7A, B). Serpina3n knockdown also inhibited the SE-induced increase in protein expression levels, whereas Serpina3n overexpression had the opposite effect (Additional file 1: Fig. S7A, B). In addition, multiple studies have shown that triptolide significantly decreased the seizure severity and reduced the glial activation and apoptosis of neurons by inhibiting NF-κB [45–48]. These results suggest that SerpinA3N exerts a proinflammatory effect by activating the NF-κB signaling pathway.

### SerpinA3N interacts with ryanodine receptor 2 (RYR2) and elevates its protein level

**Fig. 7:**
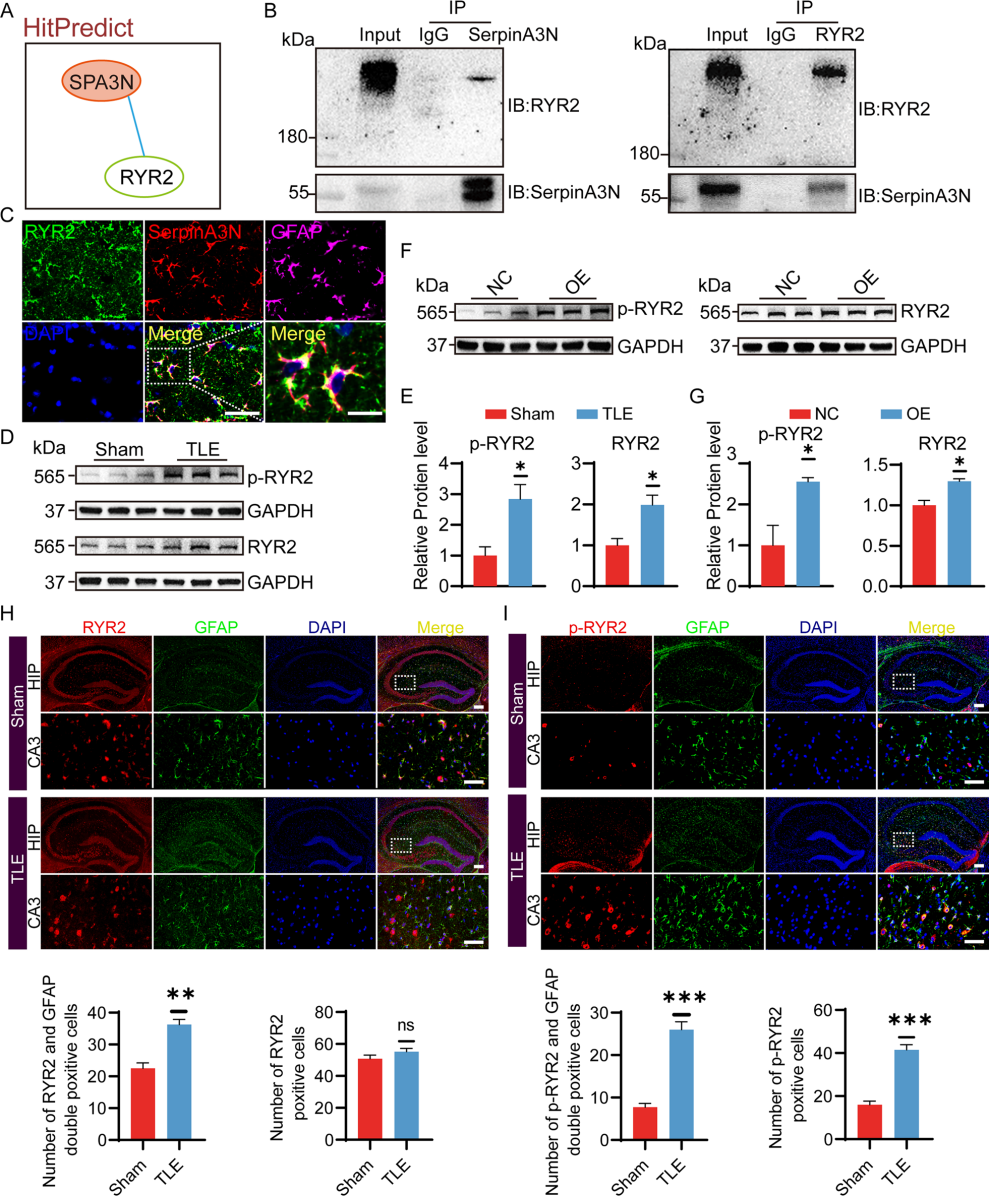
SerpinA3N interacts with RYR2 and promotes its protein levels. **A** PPI network constructed by the HitPredict website. **B** Detection of the interaction between SerpinA3N and RYR2 by Co-IP. **C** Representative fluorescence micrographs of RYR2, SerpinA3N, and GFAP expression in the hippocampal CA3 region of mice at 1 day after KA injection (scale bar (left) = 50 μm. Scale bar (right) = 20 μm). **D**, **E** Western blotting and densitometric quantitative analysis for RYR2 and p-RYR2 (S2808) in the hippocampus of mice at 35 d after KA injection. GAPDH served as the internal control (*n* = 3). **F**, **G** Western blotting and densitometric quantitative analysis of RYR2 and p-RYR2 (S2808) in the hippocampus of the SerpinA3N-overexpressing mice at 7 d post-SE. GAPDH served as the internal control (*n* = 3). **H**, **I** Dual immunofluorescence analysis with anti-RYR2 (red), anti-p-RYR2 (red) and anti-GFAP antibodies (green) in hippocampal astrocytes of sham and epileptic mice at 35 d after KA injection. (Scale bar = 50 μm; *n* = 3). All data are shown as the mean ± SEM. *p < 0.05, **p < 0.01, ***p < 0.001 versus the sham group. PPI: protein–protein interaction; Co-IP: co-immunoprecipitation

To further understand the mechanism of SerpinA3N in epilepsy, we predicted the proteins that interact with SerpinA3N through the HitPredict database (http://www.hitpredict.org/). The results showed that only RYR2 might interact with SerpinA3N (Fig. 7A), which was confirmed in mice at 1 day after SE by Co-IP (Fig. 7B).

Immunofluorescence double-staining results confirmed that SerpinA3N and RYR2 were colocalized in hippocampal astrocytes at 1 day after SE (Fig. 7C). As shown in Fig. 7D–G, the protein level of RYR2 and p-RYR2 (S2808) were increased in the mice at 35 d after SE and at 14 d after overexpression of SerpinA3N without KA treatment. Immunofluorescence staining was performed on the hippocampi of mice 35 days after KA injection using antibodies against RYR2 or p-RYR2, and GFAP or NeuN (Fig. 7H, I and Additional file 1: Figs. S8–10). The results showed a significant increase in double-positive cells for RYR2^+^ and GFAP^+^ in various regions of the hippocampus, but a decrease in double-positive cells for RYR2^+^ and NeuN^+^. However, the total number of RYR2^+^-positive cells did not change significantly. Additionally, there was a significant increase in the number of p-RYR2^+^ and GFAP^+^ double-positive cells, a decrease in p-RYR2^+^ and NeuN^+^ double-positive cells in CA3 and CA1, and a slight increase in the DG area. This may be associated with an increase in astrocytes and a decrease in neurons during epilepsy. Furthermore, it is noteworthy that p-RYR2 is predominantly expressed in astrocytes as compared to neurons. These results suggest an interaction between SerpinA3N and RYR2 in astrocytes and SerpinA3N activated RYR2 protein phosphorylation.

## Discussion

In this study, we performed transcriptomics and proteomics analysis on the hippocampus of mice with TLE, and identified that SerpinA3N was significantly upregulated in TLE. SerpinA3N promoted hippocampal inflammation and activation of astrocytes and microglia in epileptic mice and aggravated seizures and neuronal loss. This effect may occur through the activation of the NF-κB signaling pathway and RYR2 protein phosphorylation to promote hippocampal inflammation and glial cell activation. Our findings reveal a new mechanism by which SerpinA3N mediates the activation of epileptic glial cells and exacerbates inflammation in patients with epilepsy and provide a basis for developing strategies based on reducing inflammation to prevent the development of epilepsy.

Here, we first reported the abnormal transcript and protein profiles of the hippocampus in the chronic phase of TLE in mice and obtained 19 differentially expressed molecules with the same expression trend at the protein and gene level. Among them, such as FLNC and FLNA, have been reported in our previous work [49]. SerpinA3N was significantly dysregulated in the KA 35 d mice. It is worth noting that there is no linear relationship between the number of transcripts from the same locus and the number of proteins. In addition to the quantitative regulation of transcripts, there are many other important regulation pathways in the expression level of protein. In this study, SerpinA3N is upregulated by 1.53-fold at the mRNA level and 19.75-fold at the protein level. The expression value of SerpinA3N in GSE73878 and GSE122228 increased by less than 1.5-fold, which is similar to our transcriptomic analysis. In the central nervous system, astrocytes serve as the primary source of SerpinA3N, which has been identified as a marker for reactive astrocytes, where its expression is upregulated by IL-1, TNF, oncostatin M, IL-6 soluble, and IL-6 receptor complexes [21, 27, 50]. Physiologically, the basal level of SerpinA3 expression in the brain is very low, but immunohistochemical analysis reveals the presence of SerpinA3 in activated astrocytes during aging, both in human and monkeys [51, 52]. In the pathological process of epilepsy, astrocytes are abnormally activated to become reactive astrocytes. In addition, under the stimulation of inflammatory factors, reactive astrocytes will produce and secrete a large amount of SerpinA3N. Therefore, compared with the physiological state, the increase of SerpinA3N protein level in the brain tissue of epileptic mice is abnormally significant. Previous studies have shown that SerpinA1 has anti-inflammatory activity and can be quickly triggered in response to inflammation in the body [53–56]. It can also reduce the production of proinflammatory factors such as TNF-a and IL-8 [57, 58]. During inflammation, circulating levels can increase by up to threefold for the SerpinA1 and by 4- to 5-fold for the SerpinA3 [59]. Based on previous research findings, we think that in the pathological changes of epilepsy, the activation of astrocytes and the stimulation of inflammatory factors result in an upregulation of SerpinA3N expression, which plays a more dominant role in promoting neuroinflammation compared to the anti-inflammatory effects of SerpinA1. However, further research is needed to confirm this hypothesis.

SerpinA3N has been reported in a large number of studies. Relatively consistent, the expression of SerpinA3N or SerpinA3 was upregulated in aging mice and mice with pineal inflammation, diabetes, traumatic brain injury, Alzheimer's disease, aneurysmal subarachnoid hemorrhage, glioma, colon cancer and other diseases [21, 26–32, 60–62]. Our results showed that the expression level of SerpinA3N increased in TLE mice and patients with TLE. SerpinA3N, as a marker of reactive astrocytes, has been controversial [27]. Xi et al. found that SerpinA3N was only expressed in astrocytes of the hippocampus in TMT-induced brain injury [35]. However, recent studies have shown that SerpinA3N is also expressed in neurons and oligodendrocytes [27, 63, 64]. In our study, SerpinA3N was expressed mainly in hippocampal astrocytes, although we could also detect a minor expression of the protein in microglia, oligodendrocytes and neurons in the hippocampus of mice and patients with TLE and was expressed in almost all astrocytes. To our knowledge, this is the first study to explore the temporal and spatial expression patterns of SerpinA3N in epilepsy. We hypothesized that SerpinA3N may serve as a potential biomarker for epilepsy and play an important role in epilepsy.

Neuroinflammation is an intrinsic brain response involving innate immune mechanisms that activate glial, neuronal and microvascular systems [65–67]. Brain inflammation might contribute to the onset and perpetuation of seizures in a variety of epilepsies [65]. In animal models of epilepsy, targeted specific anti-inflammatory therapy has anti-epileptic activity and can ameliorate disease [66, 68]. Interestingly, SerpinA3N/SerpinA3 has opposite effects in different diseases. For example, SerpinA3N accelerates tissue repair in a diabetic mouse model of delayed wound healing [62], relieves neuronal apoptosis and the impairment of spatial learning and memory function in mice after hippocampal stab injury [29], and attenuates neuropathic pain by inhibiting T cell-derived leukocyte elastase [69]. These findings demonstrate the key role of SerpinA3N in neuroprotection. In addition, some studies have shown that elevated expression of SerpinA3N can aggravate tissue damage and show poor prognosis. For example, SerpinA3 knockdown in vitro showed decreased glioma or colon cancer cell proliferation, invasion, and migration, and its expression showed a positive correlation with poor patient prognosis [26, 30, 60, 70]. Hypothalamic inflammation is thought to contribute to obesity, and diet-induced obesity markedly increases hypothalamic expression of SerpinA3N [28]. To our knowledge, the function and regulatory mechanism of SerpinA3N in epilepsy have not been elucidated.

Our current study also found elevated expression of proinflammatory cytokines, including TNF-α, IL-1β, IL-18, IL-6 and NF-κB in mice with TLE. This result is consistent with our previous findings [36]. The expression of proinflammatory cytokines was more significant increased in TLE mice after overexpression of SerpinA3N. Neuroinflammation can increase the number of glial cell [71, 72], astrocytes in the hippocampus of epileptic rats are activated in latency and chronic states, and astrocyte cell bodies and processes are severely hypertrophic [36]. SerpinA3N has long been considered a marker of reactive astrocytes, although this is controversial. In this study, immunofluorescence staining showed that SerpinA3N significantly increases the number of astrocytes and microglia. Histopathological features of HS most commonly described in mesial TLE-HS patients include severe neuronal loss, mainly in the CA1 and CA4 hippocampal subregions, and reactive glial proliferation [36, 73]. Behavioral analysis and Nissl staining showed that SerpinA3N promoted SRS severity and aggravated hippocampal neuron loss in mice with TLE. Overexpression of SerpinA3N alone can cause the release of a large number of inflammatory factors, so it can increase the number of astrocytes and microglia and aggravate neuron loss.

The mechanism by which SerpinA3N promotes the inflammatory response is not clear. SerpinA3N upregulated IL-5, IL-13 and IL-4 in bronchoalveolar lavage fluid and lung tissues of asthmatic mice [34] and promoted the expression of CCL2 and CXCL10 and induced an increase in the number of reactive astrocytes and the expression of proinflammatory cytokines [35]. Interestingly, SerpinA3N expression in N42 neurons was upregulated by palmitic acid, leptin, as well as proinflammatory cytokines, and be blocked by the NF-κB inhibitor BAY11 [28]. These results indicate that there may be positive feedback between proinflammatory cytokines and SerpinA3N. Consistent with previous studies [74, 75], we found that the key proteins of the NF-κB signaling pathway were significantly activated in mice with TLE or in mice overexpressing SerpinA3N without KA treatment. In epileptic mice after NF-κB inhibitor treatment or pretreatment with SerpinA3N knockdown, the expression levels of proinflammatory cytokines were decreased. These results suggest that SerpinA3N promotes the release of proinflammatory cytokine by activating the NF-κB signaling pathway.

In addition, we confirmed the interaction between SerpinA3N and RYR2 through Co-IP, and immunofluorescence staining showed that SerpinA3N and RYR2 had co-localization in astrocyte cytoplasm. SerpinA3N increased the total protein levels of RYR2 and p-RYR2 (S2808). RYR2 regulates intracellular calcium concentration and is also an important regulator of Ca2^+^ signal transduction and Ca2^+^-dependent function in immune cells, which is used to regulate the human immune response [10, 76]. Dantrolene, a selective ryanodine receptor antagonist, could prevent pentylenetetrazole-induced seizures in mice by reducing the phosphorylation of RYR2 [77], which led to an increase in inflammation levels [78]. Knockdown of RYR2 also inhibited the increase in proinflammatory cytokines, including IL-1β, IL-6, and TNF-α, in rats with asthma and spinal cord injury [79, 80]. Heliox preconditioning could reduce cytoplasmic Ca2^+^ elevation by downregulating p-RYR2, thereby inhibiting necroptosis in the brain and exerting neuroprotective effects on hypoxic–ischemic encephalopathy [81]. However, loss of RYR2 has been shown to impair dendritic spine remodeling dependent on neuronal activity and trigger compensatory neuronal hyperexcitability [75], which also seems to be related to the mechanism of seizures. In our study, we found that the protein levels of RYR2 and p-RYR2 (S2808) increased in mice with TLE or overexpressing SerpinA3N without KA treatment. Double-positive cells for RYR2^+^ and GFAP^+^ in various regions of the hippocampus, but a decrease in double-positive cells for RYR2^+^ and NeuN^+^. Additionally, the number of p-RYR2^+^ and GFAP^+^ double-positive cells was increased in hippocampus of TLE mice. Furthermore, it is noteworthy that p-RYR2 is predominantly expressed in astrocytes as compared to neurons. Based on the above results, we believe that SerpinA3N interacts with RYR2 and promotes inflammation by activating RYR2 phosphorylation.

**Fig. 8:**
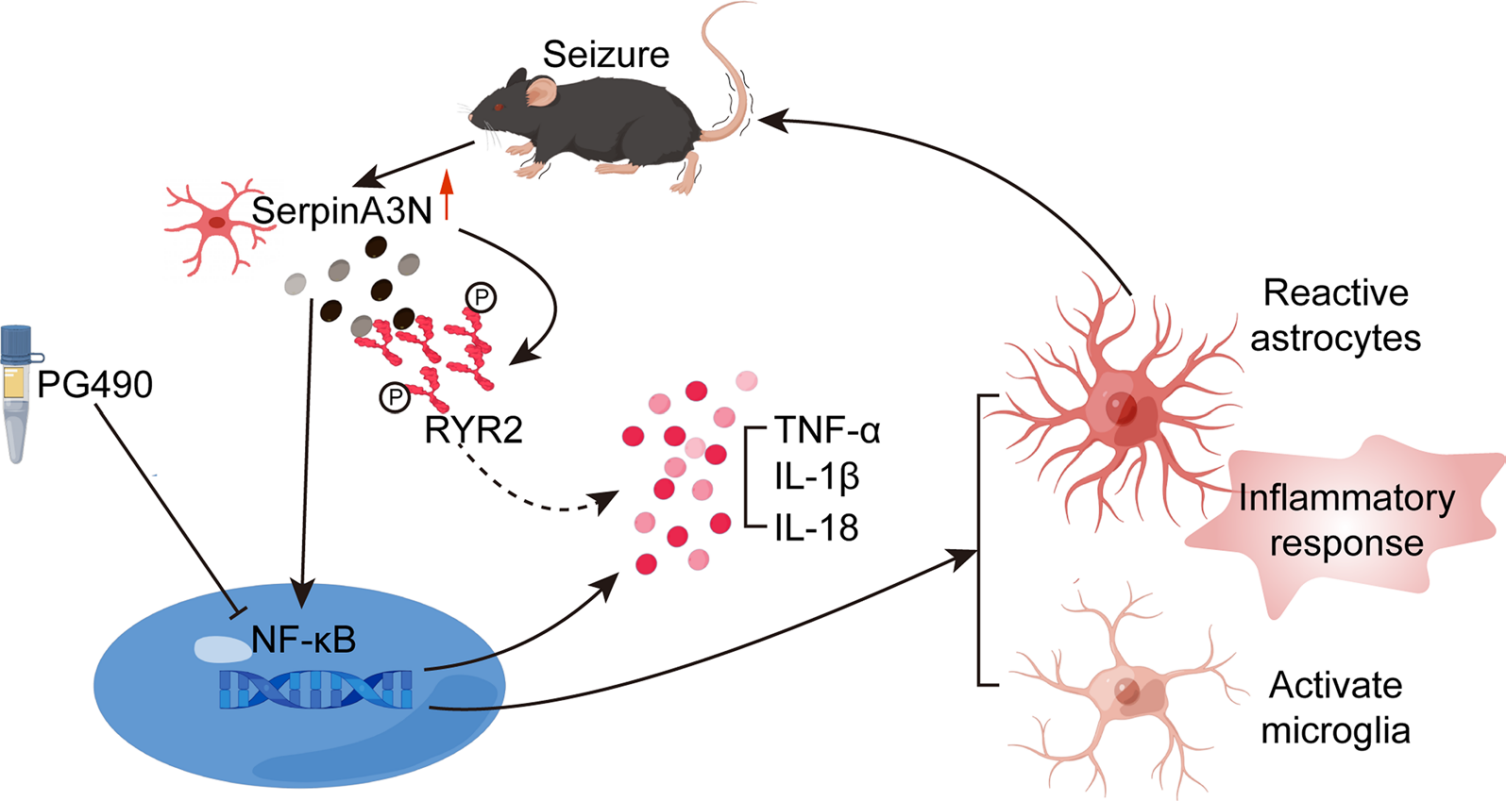
A schematic diagram showing the number of glial cells and pro-inflammation effects of SerpinA3N. Schematic model showing astrocyte-derived SerpinA3N promoting neuroinflammation and seizures by activating NF-κB signaling pathway in epileptic mice with KA-induced

Overall, these results suggest that increased SerpinA3N expression in epileptic mice leads to increased hippocampal neuroinflammation by activating the NF-κB signaling pathway and RYR2 phosphorylation (Fig. 8). SerpinA3N is often considered as a marker of reactive astrocytes, but this study found that SerpinA3N was expressed in four neuronal cell types in the hippocampus of epileptic mice. Specific manipulation of SerpinA3N in astrocytes can aggravate nerve injury and neuroinflammation, which is similar to TMT-induced neurotoxic injury, hypothalamic inflammation, and glioma, but this study cannot exclude astrocytes whether other nerve cells have the same or opposite effect.

As the basis of living matter, protein presents different positions in different tissue cells and performs different functions. Localization of the same protein in different subcellular regions can exert different functions, and even opposite effects [82]. It has been shown in previous studies that the cellular localization of SerpinA3N in the nervous system is significantly different in different disease states. Therefore, we speculate that under different stimuli, the different localization of SerpinA3N cells may be the main reason for the functional differences, which is what we will study next. Moreover, epilepsy-induced neuroinflammation in the hippocampus is not fully understood. By inhibiting SerpinA3N-mediated neuroinflammation, reducing the number of glial cells and the release of proinflammatory cytokines may be an effective means to inhibit the pathological progression of epilepsy.

## Supplementary Information

**Additional file 1.** Supplementary materials and methods. Additional Figures S1 to S10.

**Additional file 2: Table S1.** Differential expression genes of hippocampus in temporal lobe epilepsy mice.

**Additional file 3: Table S2.** Differential expression proteins of hippocampus in temporal lobe epilepsy mice.

**Additional file 4: Table S3.** Differential expression genes of hippocampus in mice at 14 days after AAV injection.

**Additional file 5: Table S4.** Primer sequences used for qPCR.

**Additional file 6: Table S5.** GEO datasets for the serpina3n/serpina3 expression analysis.

## Data Availability

The authors confirm that the data supporting the findings of this study are available within the article and its Supplementary material. Raw data that support the findings of this study are available from the corresponding author, upon reasonable request.

## References

[1] Beghi E, Beghi M (2020). Epilepsy, antiepileptic drugs and dementia. Curr Opin Neurol.

[2] Perucca P, Scheffer IE, Kiley M (2018). The management of epilepsy in children and adults. Med J Aust.

[3] Thijs RD, Surges R, O'Brien TJ, Sander JW (2019). Epilepsy in adults. Lancet.

[4] Kobow K, Auvin S, Jensen F, Loscher W, Mody I, Potschka H, Prince D, Sierra A, Simonato M, Pitkanen A (2012). Finding a better drug for epilepsy: antiepileptogenesis targets. Epilepsia.

[5] Pitkanen A, Lukasiuk K (2011). Mechanisms of epileptogenesis and potential treatment targets. Lancet Neurol.

[6] Bascunana P, Brackhan M, Leiter I, Keller H, Jahreis I, Ross TL, Bengel FM, Bankstahl M, Bankstahl JP (2020). Divergent metabolic substrate utilization in brain during epileptogenesis precedes chronic hypometabolism. J Cereb Blood Flow Metab.

[7] Chen QL, Xia L, Zhong SP, Wang Q, Ding J, Wang X (2020). Bioinformatic analysis identifies key transcriptome signatures in temporal lobe epilepsy. CNS Neurosci Ther.

[8] Bergmann C, Zerres K, Senderek J, Rudnik-Schoneborn S, Eggermann T, Hausler M, Mull M, Ramaekers VT (2003). Oligophrenin 1 (OPHN1) gene mutation causes syndromic X-linked mental retardation with epilepsy, rostral ventricular enlargement and cerebellar hypoplasia. Brain.

[9] Prontera P, Sarchielli P, Caproni S, Bedetti C, Cupini LM, Calabresi P, Costa C (2018). Epilepsy in hemiplegic migraine: Genetic mutations and clinical implications. Cephalalgia.

[10] Ma MG, Liu XR, Wu Y, Wang J, Li BM, Shi YW, Su T, Li B, Liu DT, Yi YH, Liao WP (2021). RYR2 mutations are associated with benign epilepsy of childhood with centrotemporal spikes with or without arrhythmia. Front Neurosci.

[11] Della Mina E, Ciccone R, Brustia F, Bayindir B, Limongelli I, Vetro A, Iascone M, Pezzoli L, Bellazzi R, Perotti G (2015). Improving molecular diagnosis in epilepsy by a dedicated high-throughput sequencing platform. Eur J Hum Genet.

[12] Walker A, Russmann V, Deeg CA, von Toerne C, Kleinwort KJH, Szober C, Rettenbeck ML, von Ruden EL, Goc J, Ongerth T (2016). Proteomic profiling of epileptogenesis in a rat model: focus on inflammation. Brain Behav Immun.

[13] Okamoto OK, Janjoppi L, Bonone FM, Pansani AP, da Silva AV, Scorza FA, Cavalheiro EA (2010). Whole transcriptome analysis of the hippocampus: toward a molecular portrait of epileptogenesis. BMC Genomics.

[14] Bot AM, Debski KJ, Lukasiuk K (2013). Alterations in miRNA levels in the dentate gyrus in epileptic rats. PLoS ONE.

[15] Kalozoumi G, Kel-Margoulis O, Vafiadaki E, Greenberg D, Bernard H, Soreq H, Depaulis A, Sanoudou D (2018). Glial responses during epileptogenesis in Mus musculus point to potential therapeutic targets. PLoS ONE.

[16] Liu XY, Yang JL, Chen LJ, Zhang Y, Yang ML, Wu YY, Li FQ, Tang MH, Liang SF, Wei YQ (2008). Comparative proteomics and correlated signaling network of rat hippocampus in the pilocarpine model of temporal lobe epilepsy. Proteomics.

[17] Pires G, Leitner D, Drummond E, Kanshin E, Nayak S, Askenazi M, Faustin A, Friedman D, Debure L, Ueberheide B (2021). Proteomic differences in the hippocampus and cortex of epilepsy brain tissue. Brain Commun.

[18] Mirza N, Vasieva O, Marson AG, Pirmohamed M (2011). Exploring the genomic basis of pharmacoresistance in epilepsy: an integrative analysis of large-scale gene expression profiling studies on brain tissue from epilepsy surgery. Hum Mol Genet.

[19] Debski KJ, Ceglia N, Ghestem A, Ivanov AI, Brancati GE, Broer S, Bot AM, Muller JA, Schoch S, Becker A (2020). The circadian dynamics of the hippocampal transcriptome and proteome is altered in experimental temporal lobe epilepsy. Sci Adv.

[20] Sharma S, Sharma M, Rana AK, Joshi R, Swarnkar MK, Acharya V, Singh D (2021). Deciphering key regulators involved in epilepsy-induced cardiac damage through whole transcriptome and proteome analysis in a rat model. Epilepsia.

[21] Zattoni M, Mearelli M, Vanni S, Colini Baldeschi A, Tran TH, Ferracin C, Catania M, Moda F, Di Fede G, Giaccone G (2022). Serpin signatures in prion and Alzheimer's diseases. Mol Neurobiol.

[22] Soman A, Asha Nair S (2022). Unfolding the cascade of SERPINA3: inflammation to cancer. Biochim Biophys Acta Rev Cancer.

[23] Forsyth S, Horvath A, Coughlin P (2003). A review and comparison of the murine alpha1-antitrypsin and alpha1-antichymotrypsin multigene clusters with the human clade A serpins. Genomics.

[24] Horvath AJ, Forsyth SL, Coughlin PB (2004). Expression patterns of murine antichymotrypsin-like genes reflect evolutionary divergence at the Serpina3 locus. J Mol Evol.

[25] Ma X, Niu X, Zhao J, Deng Z, Li J, Wu X, Wang B, Zhang M, Zhao Y, Guo X (2022). Downregulation of Sepina3n aggravated blood-brain barrier disruption after traumatic brain injury by activating neutrophil elastase in mice. Neuroscience.

[26] Norton ES, Da Mesquita S, Guerrero-Cazares H (2021). SERPINA3 in glioblastoma and Alzheimer's disease. Aging (Albany NY).

[27] Zhang Y, Chen Q, Chen D, Zhao W, Wang H, Yang M, Xiang Z, Yuan H (2022). SerpinA3N attenuates ischemic stroke injury by reducing apoptosis and neuroinflammation. CNS Neurosci Ther.

[28] Sergi D, Campbell FM, Grant C, Morris AC, Bachmair EM, Koch C, McLean FH, Muller A, Hoggard N, de Roos B (2018). SerpinA3N is a novel hypothalamic gene upregulated by a high-fat diet and leptin in mice. Genes Nutr.

[29] Wang ZM, Liu C, Wang YY, Deng YS, He XC, Du HZ, Liu CM, Teng ZQ (2020). SerpinA3N deficiency deteriorates impairments of learning and memory in mice following hippocampal stab injury. Cell Death Discov.

[30] Yuan Q, Wang SQ, Zhang GT, He J, Liu ZD, Wang MR, Cai HQ, Wan JH (2021). Highly expressed of SERPINA3 indicated poor prognosis and involved in immune suppression in glioma. Immun Inflamm Dis.

[31] Aslam MS, Yuan L (2020). Serpina3n: potential drug and challenges, mini review. J Drug Target.

[32] Haile Y, Carmine-Simmen K, Olechowski C, Kerr B, Bleackley RC, Giuliani F (2015). Granzyme B-inhibitor serpina3n induces neuroprotection in vitro and in vivo. J Neuroinflamm.

[33] Liu Z, Liu R, Wang R, Dai J, Chen H, Wang J, Li X (2022). Sinensetin attenuates IL-1beta-induced cartilage damage and ameliorates osteoarthritis by regulating SERPINA3. Food Funct.

[34] Zhang HT, Wang P, Li Y, Bao YB (2021). SerpinA3n affects ovalbumin (OVA)-induced asthma in neonatal mice via the regulation of collagen deposition and inflammatory response. Respir Physiol Neurobiol.

[35] Xi Y, Liu M, Xu S, Hong H, Chen M, Tian L, Xie J, Deng P, Zhou C, Zhang L (2019). Inhibition of SERPINA3N-dependent neuroinflammation is essential for melatonin to ameliorate trimethyltin chloride-induced neurotoxicity. J Pineal Res.

[36] Han CL, Ge M, Liu YP, Zhao XM, Wang KL, Chen N, Meng WJ, Hu W, Zhang JG, Li L, Meng FG (2018). LncRNA H19 contributes to hippocampal glial cell activation via JAK/STAT signaling in a rat model of temporal lobe epilepsy. J Neuroinflamm.

[37] Solomonia R, Nozadze M, Kuchiashvili N, Bolkvadze T, Kiladze M, Zhvania M, Kigyradze T, Pkhakadze V (2007). Effect of myo-inositol on convulsions induced by pentylenetetrazole and kainic acid in rats. Bull Exp Biol Med.

[38] Foust KD, Nurre E, Montgomery CL, Hernandez A, Chan CM, Kaspar BK (2009). Intravascular AAV9 preferentially targets neonatal neurons and adult astrocytes. Nat Biotechnol.

[39] Vagner T, Dvorzhak A, Wojtowicz AM, Harms C, Grantyn R (2016). Systemic application of AAV vectors targeting GFAP-expressing astrocytes in Z-Q175-KI Huntington's disease mice. Mol Cell Neurosci.

[40] Young D, Fong DM, Lawlor PA, Wu A, Mouravlev A, McRae M, Glass M, Dragunow M, During MJ (2014). Adenosine kinase, glutamine synthetase and EAAT2 as gene therapy targets for temporal lobe epilepsy. Gene Ther.

[41] Samaranch L, Salegio EA, San Sebastian W, Kells AP, Foust KD, Bringas JR, Lamarre C, Forsayeth J, Kaspar BK, Bankiewicz KS (2012). Adeno-associated virus serotype 9 transduction in the central nervous system of nonhuman primates. Hum Gene Ther.

[42] Bauer A, Puglisi M, Nagl D, Schick JA, Werner T, Klingl A, El Andari J, Hornung V, Kessler H, Gotz M (2022). Molecular signature of astrocytes for gene delivery by the synthetic adeno-associated viral vector rAAV9P1. Adv Sci (Weinh).

[43] Du T, Chen Y, Shi L, Liu D, Liu Y, Yuan T, Zhang X, Zhu G, Zhang J (2021). Deep brain stimulation of the anterior nuclei of the thalamus relieves basal ganglia dysfunction in monkeys with temporal lobe epilepsy. CNS Neurosci Ther.

[44] Hayatdavoudi P, Hosseini M, Hajali V, Hosseini A, Rajabian A (2022). The role of astrocytes in epileptic disorders. Physiol Rep.

[45] Sun Z, Du M, Lu Y, Zeng CQ (2018). Effects of triptolide on the expression of MHC II in microglia in kainic acid-induced epilepsy. Mol Med Rep.

[46] He LY, Hu MB, Li RL, Zhao R, Fan LH, He L, Lu F, Ye X, Huang YL, Wu CJ (2021). Natural medicines for the treatment of epilepsy: bioactive components. Pharmacol Mech Front Pharmacol.

[47] Jiang J, Feng J, Wu L, Liang J, He Y (2020). Li CJCTiNR: Triptolide Inhibits neuronal apoptosis in a rat model of pentylenetetrazol-induced-epilepsy via upregulation of miR-187 expression. Curr Topic Nutraceut Res..

[48] Cui Y, Jiang X, Feng J (2022). The therapeutic potential of triptolide and celastrol in neurological diseases. Front Pharmacol.

[49] Han CL, Zhao XM, Liu YP, Wang KL, Chen N, Hu W, Zhang JG, Ge M, Meng FG (2019). Gene expression profiling of two epilepsy models reveals the ECM/Integrin signaling pathway is involved in epileptogenesis. Neuroscience.

[50] Kordula T, Bugno M, Rydel RE, Travis J (2000). Mechanism of interleukin-1- and tumor necrosis factor alpha-dependent regulation of the alpha 1-antichymotrypsin gene in human astrocytes. J Neurosci.

[51] Koo EH, Abraham CR, Potter H, Cork LC, Price DL (1991). Developmental expression of alpha 1-antichymotrypsin in brain may be related to astrogliosis. Neurobiol Aging.

[52] Abraham CR, Selkoe DJ, Potter H (1988). Immunochemical identification of the serine protease inhibitor alpha 1-antichymotrypsin in the brain amyloid deposits of Alzheimer's disease. Cell.

[53] Hurley K, Lacey N, O'Dwyer CA, Bergin DA, McElvaney OJ, O'Brien ME, McElvaney OF, Reeves EP, McElvaney NG (2014). Alpha-1 antitrypsin augmentation therapy corrects accelerated neutrophil apoptosis in deficient individuals. J Immunol.

[54] Song S (2018). Alpha-1 antitrypsin therapy for autoimmune disorders. Chronic Obstr Pulm Dis.

[55] de Serres F, Blanco I (2014). Role of alpha-1 antitrypsin in human health and disease. J Intern Med.

[56] Kaneva MK, Muley MM, Krustev E, Reid AR, Souza PR, Dell'Accio F, McDougall JJ, Perretti M (2021). Alpha-1-antitrypsin reduces inflammation and exerts chondroprotection in arthritis. FASEB J.

[57] Nita IM, Serapinas D, Janciauskiene SM (2007). alpha1-Antitrypsin regulates CD14 expression and soluble CD14 levels in human monocytes in vitro. Int J Biochem Cell Biol.

[58] Janciauskiene S, Larsson S, Larsson P, Virtala R, Jansson L, Stevens T (2004). Inhibition of lipopolysaccharide-mediated human monocyte activation, in vitro, by alpha1-antitrypsin. Biochem Biophys Res Commun.

[59] Kalsheker N, Morley S, Morgan K (2002). Gene regulation of the serine proteinase inhibitors alpha1-antitrypsin and alpha1-antichymotrypsin. Biochem Soc Trans.

[60] Cao LL, Pei XF, Qiao X, Yu J, Ye H, Xi CL, Wang PY, Gong ZL (2018). SERPINA3 silencing inhibits the migration, invasion, and liver metastasis of colon cancer cells. Dig Dis Sci.

[61] Slowik A, Borratynska A, Turaj W, Pera J, Dziedzic T, Figlewicz DA, Betlej M, Krzyszkowski T, Czepko R, Szczudlik A (2005). Alpha1-antichymotrypsin gene (SERPINA3) A/T polymorphism as a risk factor for aneurysmal subarachnoid hemorrhage. Stroke.

[62] Hsu I, Parkinson LG, Shen Y, Toro A, Brown T, Zhao H, Bleackley RC, Granville DJ (2014). Serpina3n accelerates tissue repair in a diabetic mouse model of delayed wound healing. Cell Death Dis.

[63] Murphy CE, Kondo Y, Walker AK, Rothmond DA, Matsumoto M, Shannon Weickert C (2020). Regional, cellular and species difference of two key neuroinflammatory genes implicated in schizophrenia. Brain Behav Immun.

[64] Kenigsbuch M, Bost P, Halevi S, Chang Y, Chen S, Ma Q, Hajbi R, Schwikowski B, Bodenmiller B, Fu H (2022). A shared disease-associated oligodendrocyte signature among multiple CNS pathologies. Nat Neurosci.

[65] Vezzani A, French J, Bartfai T, Baram TZ (2011). The role of inflammation in epilepsy. Nat Rev Neurol.

[66] Vezzani A, Balosso S, Ravizza T (2019). Neuroinflammatory pathways as treatment targets and biomarkers in epilepsy. Nat Rev Neurol.

[67] Casillas-Espinosa PM, Ali I, O'Brien TJ (2020). Neurodegenerative pathways as targets for acquired epilepsy therapy development. Epilepsia Open.

[68] Sharma R, Leung WL, Zamani A, O'Brien TJ, Casillas Espinosa PM, Semple BD (2019). Neuroinflammation in post-traumatic epilepsy: pathophysiology and tractable therapeutic targets. Brain Sci.

[69] Vicuna L, Strochlic DE, Latremoliere A, Bali KK, Simonetti M, Husainie D, Prokosch S, Riva P, Griffin RS, Njoo C (2015). The serine protease inhibitor SerpinA3N attenuates neuropathic pain by inhibiting T cell-derived leukocyte elastase. Nat Med.

[70] Nimbalkar VP, Kruthika BS, Sravya P, Rao S, Sugur HS, Verma BK, Chickabasaviah YT, Arivazhagan A, Kondaiah P, Santosh V (2021). Differential gene expression in peritumoral brain zone of glioblastoma: role of SERPINA3 in promoting invasion, stemness and radioresistance of glioma cells and association with poor patient prognosis and recurrence. J Neurooncol.

[71] Sun L, Shan W, Yang H, Liu R, Wu J, Wang Q (2021). The role of neuroinflammation in post-traumatic epilepsy. Front Neurol.

[72] Gong L, Zhu T, Chen C, Xia N, Yao Y, Ding J, Xu P, Li S, Sun Z, Dong X (2022). Miconazole exerts disease-modifying effects during epilepsy by suppressing neuroinflammation via NF-kappaB pathway and iNOS production. Neurobiol Dis.

[73] Lentini C, d'Orange M, Marichal N, Trottmann MM, Vignoles R, Foucault L, Verrier C, Massera C, Raineteau O, Conzelmann KK (2021). Reprogramming reactive glia into interneurons reduces chronic seizure activity in a mouse model of mesial temporal lobe epilepsy. Cell Stem Cell.

[74] Guo X, Wang J, Wang N, Mishra A, Li H, Liu H, Fan Y, Liu N, Wu Z (2020). Wogonin preventive impact on hippocampal neurodegeneration, inflammation and cognitive defects in temporal lobe epilepsy. Saudi J Biol Sci.

[75] Sanz P, Garcia-Gimeno MA (2020). Reactive glia inflammatory signaling pathways and epilepsy. Int J Mol Sci.

[76] Santulli G, Pagano G, Sardu C, Xie W, Reiken S, D'Ascia SL, Cannone M, Marziliano N, Trimarco B, Guise TA (2015). Calcium release channel RyR2 regulates insulin release and glucose homeostasis. J Clin Invest.

[77] Keshavarz M, Fotouhi M, Rasti A (2016). Dantrolene: a selective ryanodine receptor antagonist, protects against pentylenetetrazole-induced seizure in mice. Acta Med Iran.

[78] Nofi C, Zhang K, Tang YD, Li Y, Migirov A, Ojamaa K, Gerdes AM, Zhang Y (2020). Chronic dantrolene treatment attenuates cardiac dysfunction and reduces atrial fibrillation inducibility in a rat myocardial infarction heart failure model. Heart Rhythm.

[79] Liao B, Zhang Y, Sun H, Ma B, Qian J (2016). Ryanodine receptor 2 plays a critical role in spinal cord injury via induction of oxidative stress. Cell Physiol Biochem.

[80] Yang M, Wang LI (2021). MALAT1 knockdown protects from bronchial/tracheal smooth muscle cell injury via regulation of microRNA-133a/ryanodine receptor 2 axis. J Biosci.

[81] Zhong W, Cheng J, Yang X, Liu W, Li Y (2022). Heliox preconditioning exerts neuroprotective effects on neonatal ischemia/hypoxia injury by inhibiting necroptosis induced by Ca(2+) elevation. Transl Stroke Res.

[82] Zhu Z, Tan J, Deng H (2019). Nucleus translocation of membrane/cytoplasm proteins in tumor cells. Zhejiang Da Xue Xue Bao Yi Xue Ban.

