# Supplementary material for: Astrocyte-derived SerpinA3N promotes neuroinflammation and epileptic seizures by activating the NF-κB signaling pathway in mice with temporal lobe epilepsy
Source: J Neuroinflammation. 2023 Jul 8;20:161. doi: 10.1186/s12974-023-02840-8 (PMC10329806; doi:10.1186/s12974-023-02840-8)
Supplement: Supplementary file 1 — Additional file 1. Supplementary materials and methods. Additional Figures S1 to S10. [file 12974_2023_2840_MOESM1_ESM.docx]

**Additional file for**

**Astrocyte-derived SerpinA3N promotes neuroinflammation and epileptic seizures by activating the NF-κB signaling pathway in mice with temporal lobe epilepsy**

**This file includes:**

1. Supplementary Materials and Methods

2. Supplementary figure and legends: figure S1 to S10

**1. Supplementary Materials and Methods**

**1.1 Transcriptome and quantitative proteomics analysis**

For the transcriptome analysis, according to the manufacturer’s standard operating procedures, the Low Input Quick Amp Labeling Kit, One-Color (Cat# 5190-2305, Agilent Technologies) was used to amplify and label total RNA from four sham tissues and four epileptic hippocampal tissues, while the RNeasy mini kit (Cat# 74106, QIAGEN, GmBH, Germany) was used to purify labeled cRNA. A gene Expression Hybridization Kit (Cat# 5188-5242, Agilent Technologies) was used for cRNA hybridization according to the manufacturer’s instructions. After the Agilent slides were washed, they were scanned with an Agilent Microarray Scanner (Cat# G2565CA, Agilent Technologies). Feature Extraction software 10.7 (Agilent Technologies) was then used to read the data. Finally, the limma package in R software was used to normalize the data, and the Quantile algorithm was used.

For the quantitative proteomics analysis, three sham group and three epileptic hippocampal tissues were ground with liquid nitrogen into cell powder and then transferred to a 5 mL centrifuge tube. Then, four volumes of lysis buffer were added to the cell powder, followed by sonication three times on ice using a high-intensity ultrasonic processor. The remaining debris was removed by centrifugation at 12,000 g at 4 °C for 10 min. Finally, the supernatant was collected, and the protein concentration was determined with a BCA kit (Beyotime Biotechnology, Nantong, China) according to the manufacturer’s instructions. The protein solution was reduced with 5 mM dithiothreitol for 30 min at 56 °C and alkylated with 11 mM iodoacetamide for 15 min at room temperature in darkness. The protein sample was then diluted by adding 100 mM TEAB to urea with a concentration less than 2 M. Finally, trypsin was added at a 1:50 trypsin-to-protein mass ratio for the first digestion overnight and a 1:100 trypsin-to-protein mass ratio for a second 4 h digestion. Finally, the peptides were desalted by a C18 SPE column. The peptides were subjected to capillary source followed by timsTOF Pro (Bruker Daltonics) mass spectrometry. The electrospray voltage applied was 1.60 kV. Precursors and fragments were analyzed at the TOF detector, with an MS/MS scan range from 100 to 1700 m/z. The timsTOF Pro was operated in parallel accumulation serial fragmentation (PASEF) mode. Precursors with charge states of 0 to 5 were selected for fragmentation, and 10 PASEF-MS/MS scans were acquired per cycle. The dynamic exclusion was set to 30 s. The tryptic peptides were dissolved in solvent A (0.1% formic acid, 2% acetonitrile/in water) and directly loaded onto a homemade reversed-phase analytical column (25 cm length, 75/100 μm i.d.). Peptides were separated with a gradient from 6% to 24% solvent B (0.1% formic acid in acetonitrile) over 70 min and 24% to 35% in 14 min, climbing to 80% in 3 min and then holding at 80% for the last 3 min, all at a constant flow rate of 450 nL/min on a nanoElute UHPLC system (Bruker Daltonics). The resulting MS/MS data were processed using the MaxQuant search engine (v.1.6.15.0). Tandem mass spectra were searched against the human SwissProt database (20422 entries) concatenated with the reverse decoy database. Trypsin/P was specified as a cleavage enzyme allowing up to 2 missing cleavages. The mass tolerance for precursor ions was set as 20 ppm in the first search and 5 ppm in the main search, and the mass tolerance for fragment ions was set as 0.02 Da. Carbamidomethyl on Cys was specified as a fixed modification, and acetylation on the protein N-terminus and oxidation on Met were specified as variable modifications. FDR was adjusted to < 1%.

**1.2** **Construction of the SerpinA3N overexpression and silencing adeno-associated virus**

For AAV9-OE packaging, the sequence of the AAV9-OE primers was 5′-ATGGACTACAAAGACG ATGACGACAAG-3′. A pAAV[Exp]-GFAP(short)>FLAG/mSerpina3n[NM_0092 52.2](ns):T2A:mCherry: WPRE vector was used and packaged with adeno-associated virus (AAV). For AAV9-shRNA packaging, the sequence of the shRNA primers was 5′-gtccaagaagaccatgacaat-3′. A pHBAAV-GFAP-MCS-P2A-mCherry was used and packaged with AAV. The negative control was an empty AAV vector (AAV-NC) or one harboring a scrambled sequence (AAV-Scr: 5′- AAGGTATATTGCTGTTGACAGTGAGCGgtccaagaagaccatgacaatTAGTGAAGCCACAGATGTAattgtcatggtcttcttggacTGCCTACTGCCTCG-3′). SerpinA3N overexpression and silencing adeno-associated virus plasmid was constructed as illustrated in Figure S4. The titers used were 1.8 × 10^13^ for AAV9-OE and 2.0 ×10^13^ for AAV9-shRNA.

**1.3** **Western blot analysis and coimmunoprecipitation (Co-IP)**

Briefly, for the Western blot procedures, tissue was washed with PBS solution and lysed in radioimmunoprecipitation lysis buffer (50 mM Tris-HCl [pH 7.4], 150 mM sodium chloride, 1% Nonidet P-40, 0.1% sodium dodecyl sulfate [SDS]) (Cat#C1053; Applygen Technologies, Inc., Beijing, China), phosphate and protease inhibitor cocktails. The samples were mixed thoroughly with an electric homogenizer on ice and allowed to react for 20 min. The homogenates were centrifuged at 12,000 × g at 4 °C for 20 min. The protein concentrations in the supernatants were determined with a Pierce^TM^ protein assay kit (Cat#23227, Thermo Scientific Pierce, USA). Then, 4-12% or 12% MOPS-PAGE gels (Beijing Lablead Biotech, Beijing, China) were loaded with 50 μg of lysate protein per well for separation, and the proteins were transferred to polyvinylidene difluoride membranes (Cat#3010040001, Millipore, USA). After the membranes were blocked with 10% milk for 1 h, they were incubated with the following primary antibodies: goat anti-SerpinA3N (RD; AF4709), mouse anti-TNF-α (Santa Cruz; sc-52746), mouse anti-IL-1β (Abcam; ab234437), rabbit anti-IL-18 (Origene; TA377434), rabbit anti-NF-κB p65 (Huabio; ET1603-12), rabbit anti-phospho-NF-κB p65 (CST; MAB3033), rabbit anti-IKKα+β (Huabio; ET1611-23), rabbit anti-phospho-IKKα+β (Abcam; ab194528), rabbit anti-GFAP (PTM Bio; PTM-6104), rabbit anti-IBA1 (Huabio; ET1705-78), rabbit anti-phospho-RYR2 (Ser2808) (Biorbyt; orb1093816), and mouse anti-RYR2 (Thermo Fisher Scientific, MA3-916). The rabbit anti-GAPDH recombinant monoclonal antibody (Huabio; ET1601-4) was used as a control. The membranes were incubated with an appropriate secondary antibody (Santa Cruz; sc-2354, LABLEAD; S0101, LABLEAD; S0100).

For the Co-IP procedures, fresh hippocampal tissue (KA 1d) was washed with prechilled sterile PBS, 300 µl of IP lysis buffer with protease inhibitor and phosphatase inhibitor was added, the tissue was disrupted using a tissue homogenizer, and the samples were sonicated 3 times on ice (25 W), 3 seconds each time. The samples were centrifuged at 12,000 x g for 20 min at 4 °C, and the supernatant was transferred to a new tube. Then, 5 μl of Protein A and 5 μl of Protein G agarose beads were added to 250 μl of cell lysate (containing 800-1000 µg total protein) and incubated at 4 °C for 60 minutes with rotation. The samples were centrifuged at 12000 × g for 1 min at 4 °C, and the supernatant was saved. Then, the corresponding antibody to the target protein and the nonspecific immunization homologous antibody (5 μg) were added, along with 5 μl of Protein A and 5 μl of Protein G, and mixed gently overnight at 4 °C. The samples were centrifuged at 12,000 × g for 1 minute to retain the pellet. The pellet was washed 3 times with 0.5 ml of 1*wash buffer and centrifuged at 12,000 × g for 1 min, and the pellet was retained. Then, 30 μl of 1*SDS sample buffer was added to resuspend the pellet, the sample was heated to 95 °C for 5 min, and then centrifuged briefly at 12,000 × g for 1 min, and the supernatant was collected. Samples (15 μl) were loaded on a 4-12% MOPS-PAGE gel, and the corresponding protein content of the samples was analyzed by Western blotting. As needed, the membrane was stripped by incubating in Restore PLUS Western blot stripping buffer (Thermo Fisher Scientific, 46430) for 30 min at room temperature. Protein band density was scanned with an Epson V330 Photo scanner (Seiko Epson Co.) or a Chemidoc imaging system (Bio-Rad) and quantified using ImageJ software.

**2. Supplementary figure and legends**


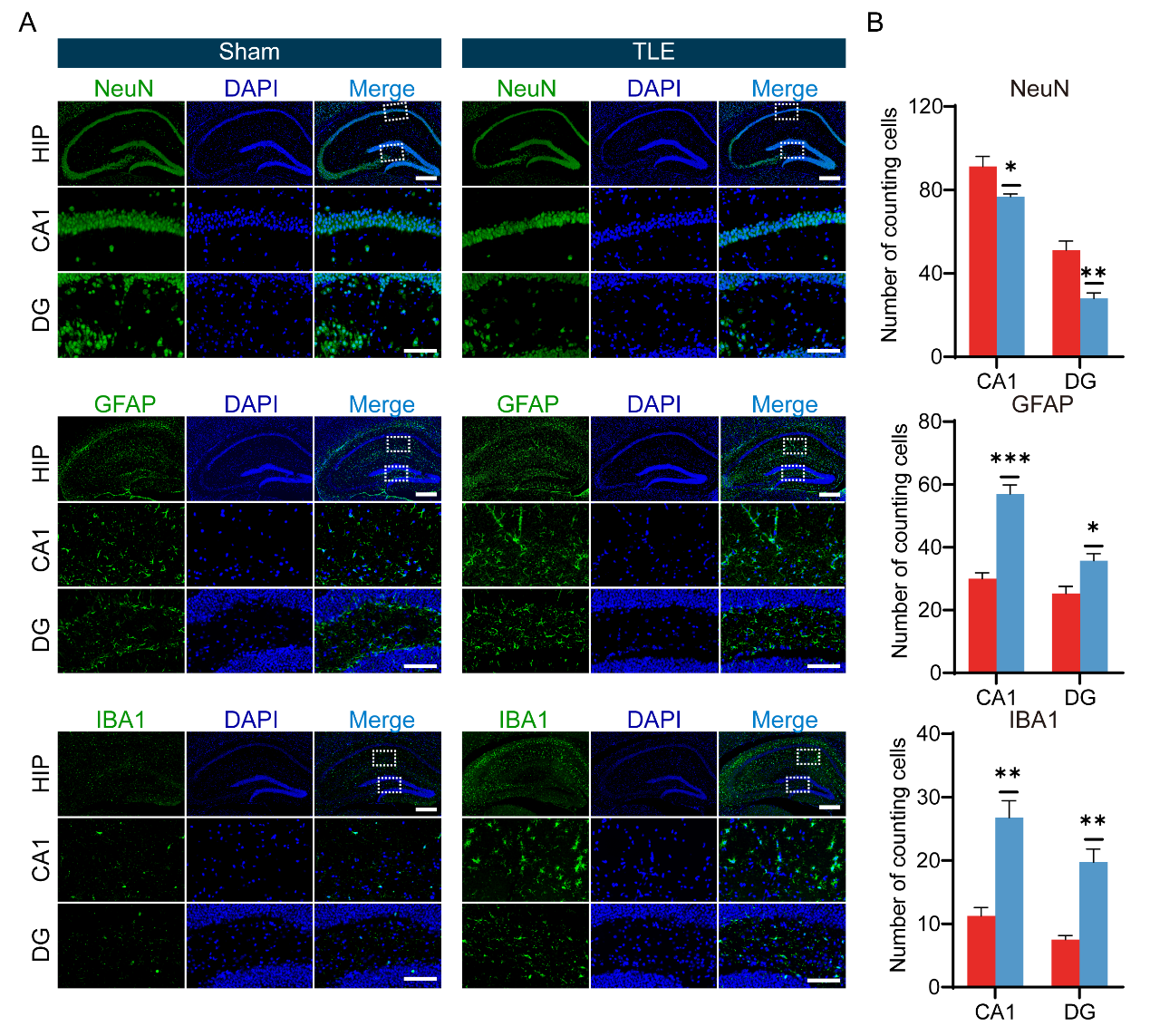
**Figure S1 Neuronal loss and increased glial cell numbers in the hippocampus of TLE mice.** (A, B) Immunostaining and quantitative analysis for NeuN, GFAP, and Iba1 cells number in the hippocampus (scale bars= 200 µm), CA1, and DG region (scale bars= 100 µm) of the hippocampus from the epileptic mice at 35 days after KA injection. (n = 4). Normalized to the sham level. All data are shown as the mean ± SEM. *P < 0.05; **P <0.01; ***P <0.001. TLE, temporal lobe epilepsy. HIP, hippocampus.

**
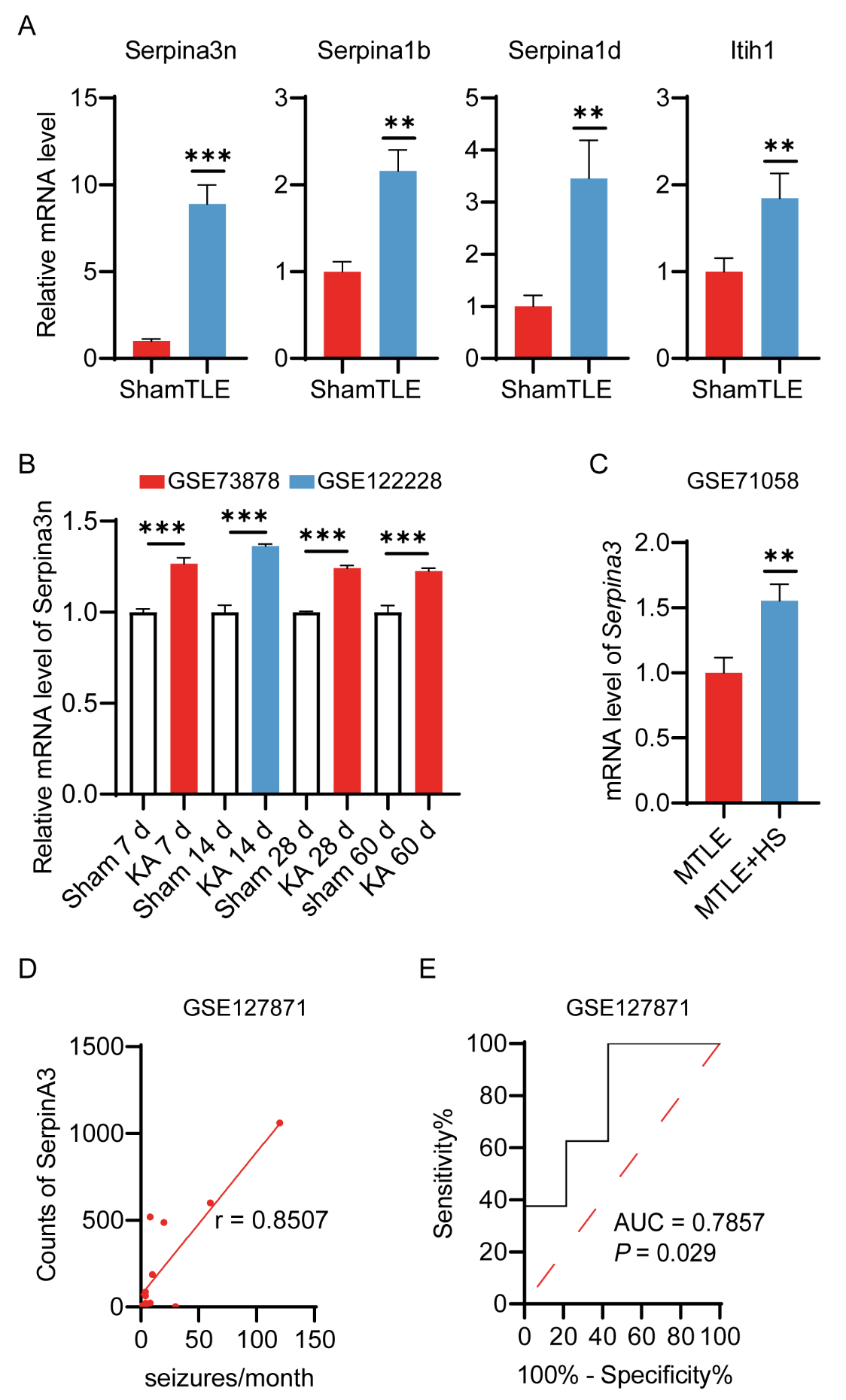
**

**Figure S2 SerpinA3N was highly expressed in the hippocampus of mice and patients with TLE.** (A) The expression of SerpinA3N, Serpina1b, Serpina1d and Itih1 was determined by qPCR in the hippocampus of epileptic mice at 35 days after KA injection (n = 4). Normalized to the sham level. Gapdh served as the internal control. (B) The expression of SerpinA3N in the hippocampus of epilepsy models (GSE73878 (n= 40), GSE122228 (n = 6)). GEO2R was used to analyze the differential expression of SerpinA3N. Normalized to the sham level. (C) SerpinA3N in the hippocampus of TLE patients with or without HS from GSE71958. Normalized to TLE patients without HS. (D) Association between the number of seizures per month and the expression level of SerpinA3 from GSE127871. (E) ROC curves confirmed that SerpinA3 levels significantly differentiated the number of seizures per month in TLE patients. All data are shown as the mean ± SEM. **P* < 0.05; ***P* <0.01; ****P* <0.001. KA, kainic acid. TLE, temporal lobe epilepsy. HS, hippocampal sclerosis. ROC, receiver operating characteristic.


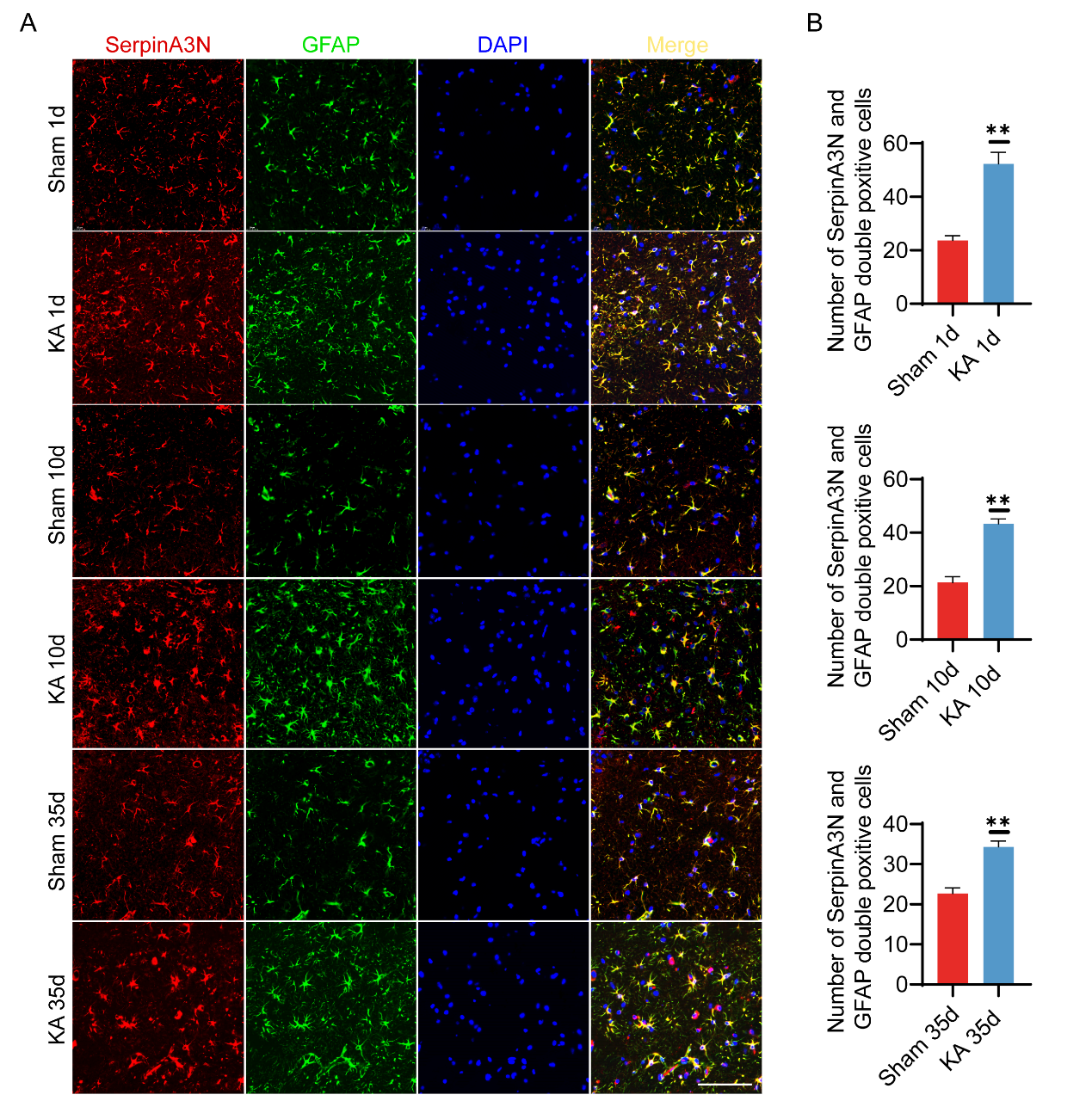


**Figure S3 The number of SerpinA3N cells increased in hippocampal astrocytes of epileptic mice.** (A, B) Immunostaining of SerpinA3N and GFAP in the hippocampal CA3 region of epileptic mice at 1, 10, and 35 d after KA injection. The right panels show the cell counts of double-positive cells with serpinA3N and GFAP (scale bar = 100 μm; n = 3). All data are shown as the mean ± SEM. *p < 0.05, **p < 0.01, ***p < 0.001. KA, kainic acid.

**
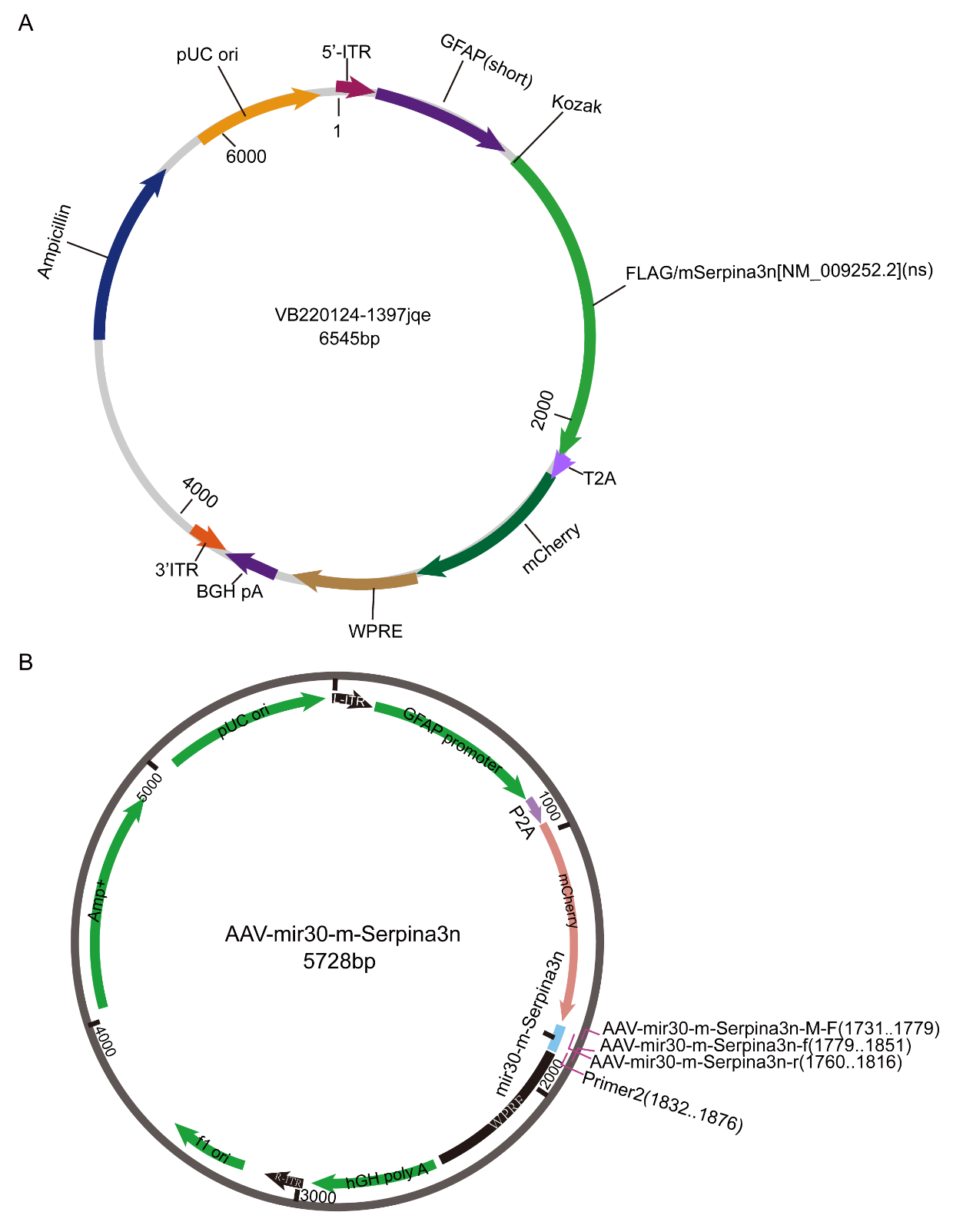
**

**Figure S4 Schematic representation of a modified AAV vector for the delivery of SerpinA3N overexpression and knockdown.** (A, B) Construction of adeno-associated virus carrying overexpression (A) and short hairpin RNA (B) for SerpinA3N. L-ITR, left adeno-associated virus (AAV)-9 inverted terminal repeat (5’); R-ITR, right AAV-2 inverted terminal repeat (3’); GFAP, GFAP promoter.


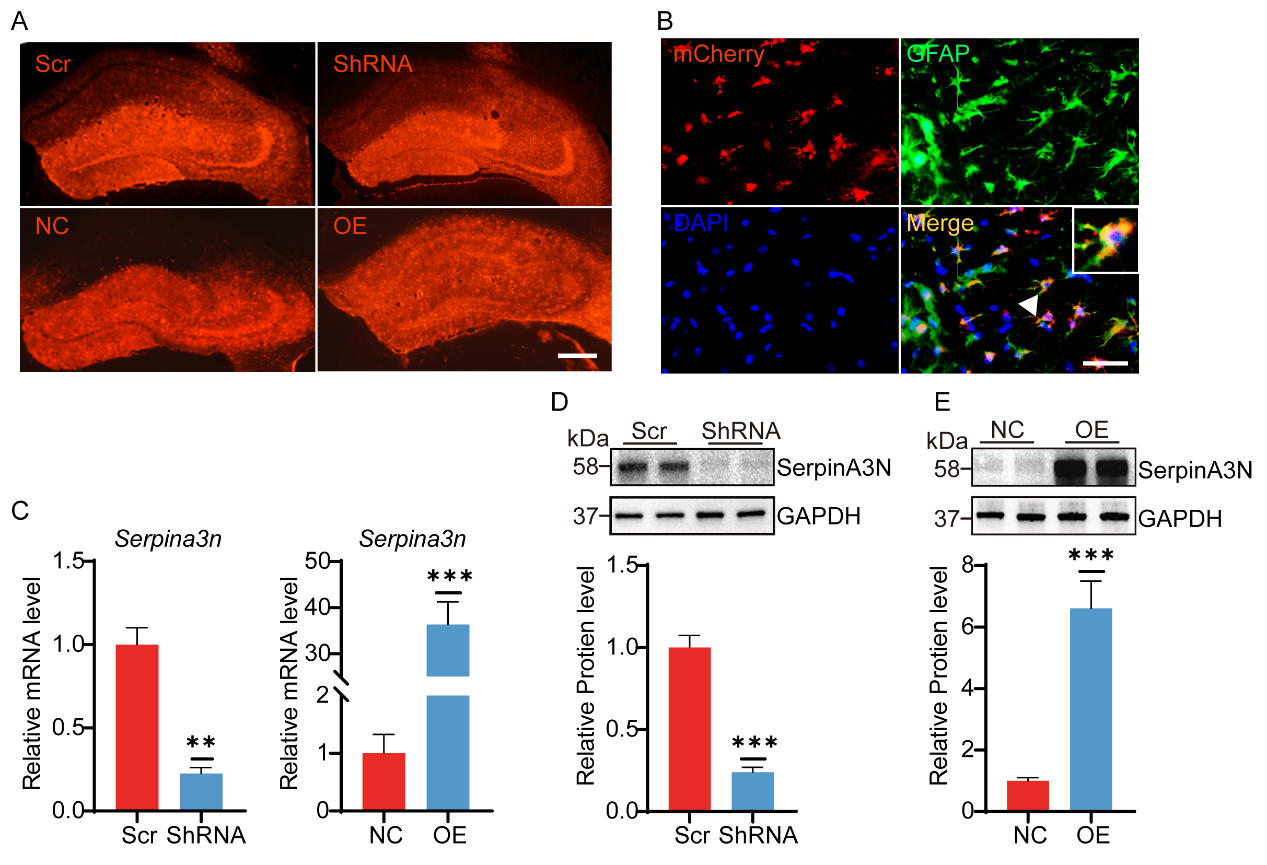


**Figure S5 Construction of the SerpinA3N overexpression and knockdown AAV delivery system specifically in astrocytes.** (A) Representative mCherry fluorescence images from mice injected in hippocampi with SerpinA3N overexpression and SerpinA3N knockdown vectors as well as their control vectors at 14 days after AAV vector injection (scale bar = 400 μm). (B) Representative fluorescence images of SerpinA3N (mCherry) and GFAP (Alexa Fluor 488) in the hippocampal CA3 region of mice at 14 days after SerpinA3N overexpression AAV vector injection (scale bar=50 μm). (C-E) Quantification of SerpinA3N expression examined by qPCR and Western blotting in the hippocampus with stereotaxic injection of NC, Scr, OE, and shRNA targeting SerpinA3N AAV vectors for 14 days (n = 4-6). Normalized to Scr or NC levels. GAPDH served as the internal control. All data are shown as the mean ± SEM. ***p* < 0.01, ****p*< 0.001. AAV, adeno-associated virus. NC, empty AAV vectors. Scr, scrambled AAV vectors. OE, AAV vectors containing overexpression SerpinA3N. ShRNA, AAV vectors containing short hairpin RNA targeting SerpinA3N.

**
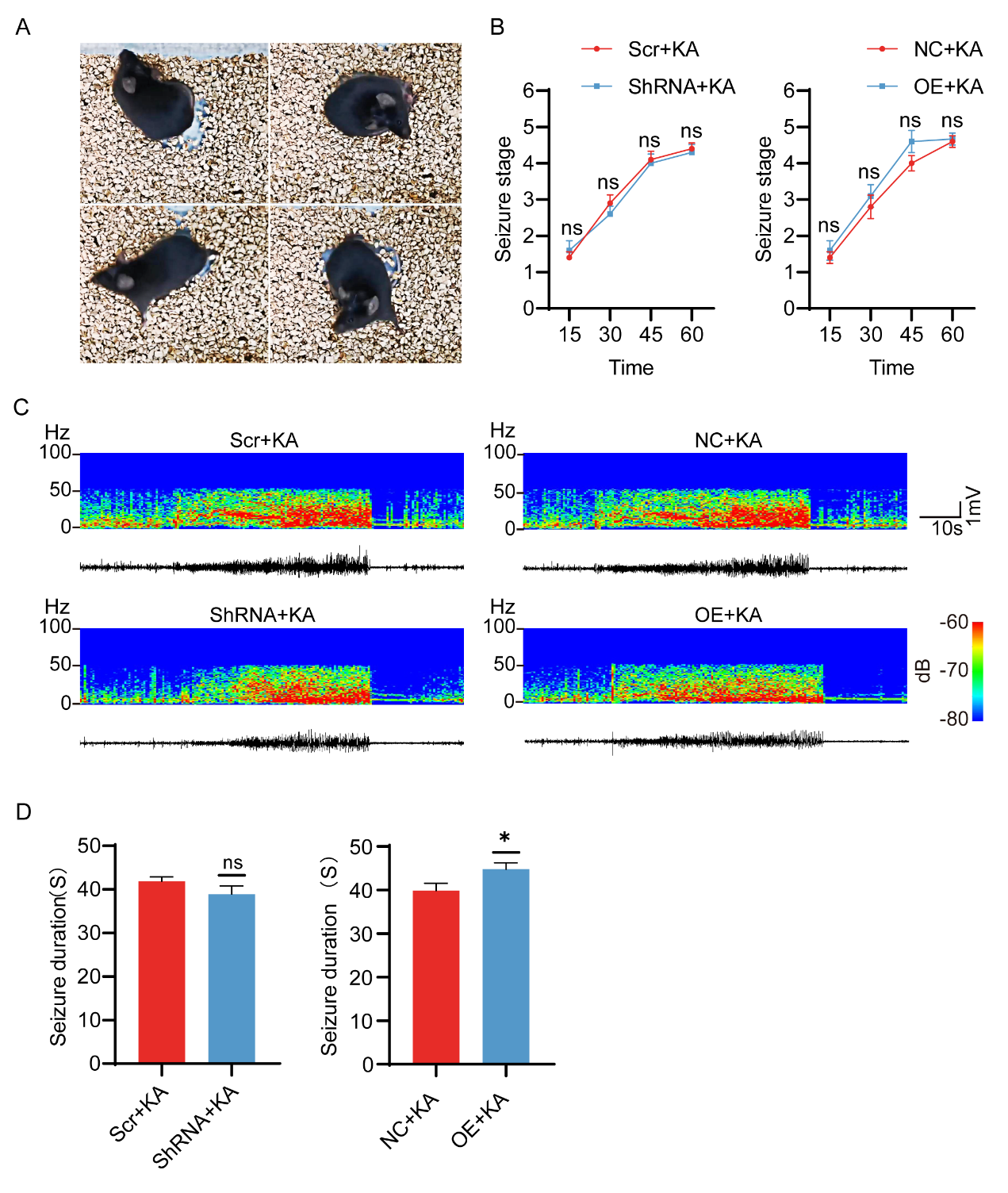
**

**Figure S6 SerpinA3N overexpression aggravated seizure duration in mice with TLE.** (A) Series of still shots exemplifying seizure behaviors in mice with status epilepticus within 1 hour of KA injection. (B) The graph shows the progression of behavioral changes over a 1 h observation period after KA injection (n = 10). (C) Upper, peri-ictal time-frequency spectrograms showing the frequency and power density before, during, and after the representative seizure. Lower panel, corresponding EEG signal during the development of seizure-like activity in differently treated epileptic mice. (D) The mean duration of seizures in mice at 28 to 35 days after KA injection. Seizures were recorded for 7 days (n = 6). All data are shown as the mean ± SEM. Ns, no significant. **p* < 0.05. KA, kainic acid. EEG, electroencephalogram.


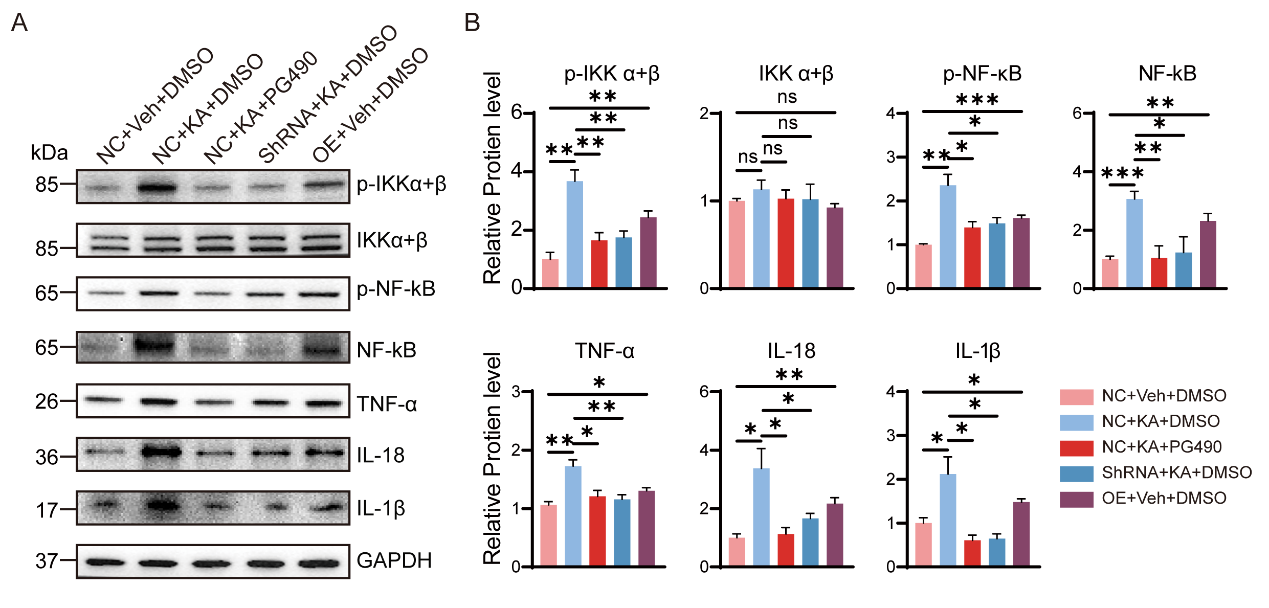


**Figure S7 Targeting the NF-κB signaling pathway inhibits the KA-induced proinflammatory response.** (A, B) Western blotting and densitometric quantitative analysis of NF-κB (p65), p-NF-κB (p65), p-IKKα+β, IKKα+β, TNF-α, IL-1β, and IL-18 in the mice in the NC, OE and shRNA groups with or without KA treatment treated with DMSO (0.5%) or PG490 (i.p., 300 µg/kg) for 7 days. GAPDH served as the internal control (n = 4). All data are shown as the mean ± SEM. ns, not significant. **p* < 0.05, ***p* < 0.01, ****p* < 0.001. NC, empty AAV vectors. OE, AAV vectors for overexpression of SerpinA3N. ShRNA, AAV vectors containing short hairpin RNA targeting SerpinA3N. KA, kainic acid. DMSO, dimethyl sulfoxide. PG490, triptolide.


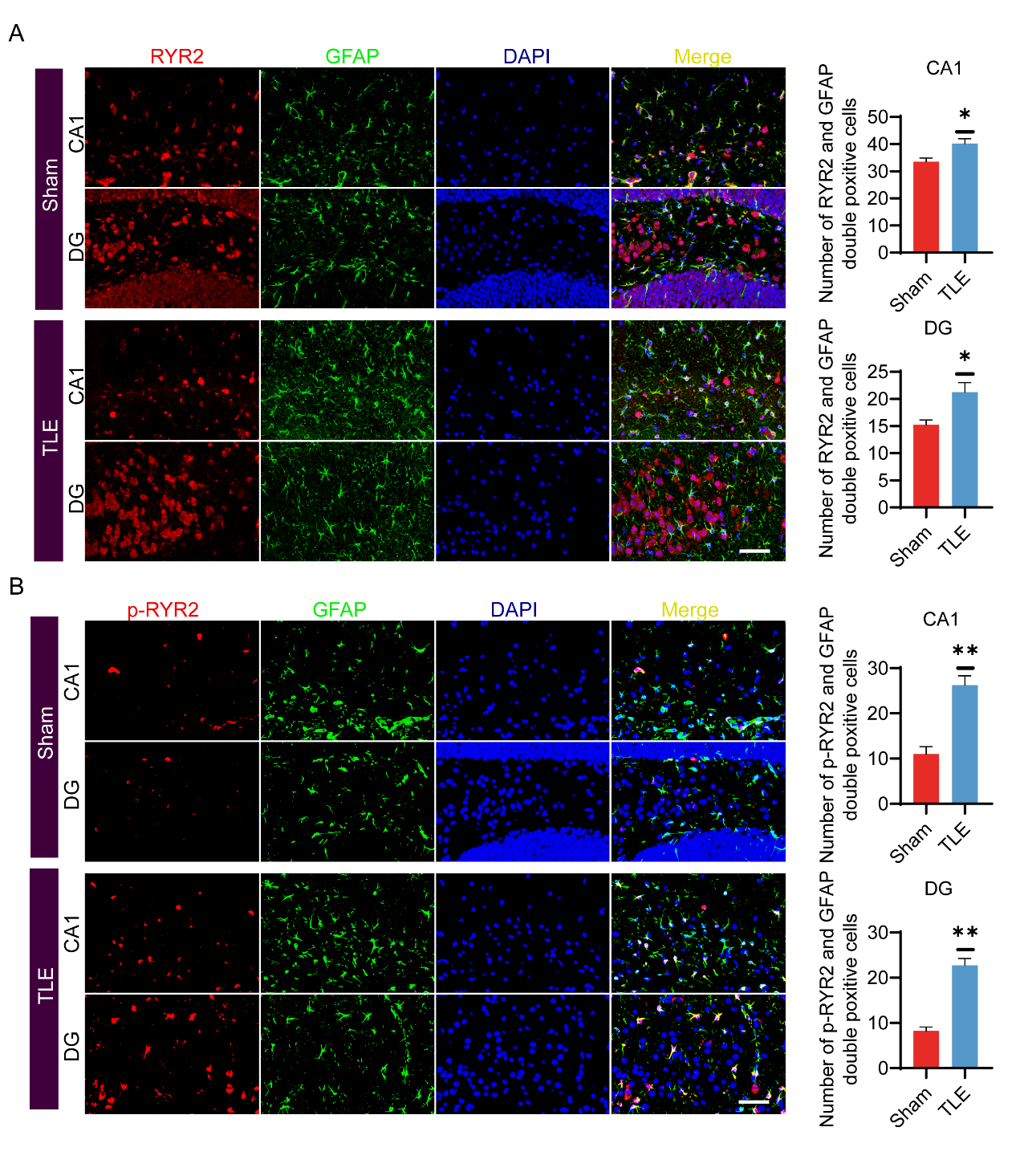


**Figure S8 Expression and activity of RYR2 in hippocampal astrocytes of epileptic mice.** (A, B) Dual immunofluorescence analysis with anti-RYR2 (red), anti-p-RYR2 (red) and anti-GFAP antibodies (green) in hippocampal astrocytes of sham and epileptic mice at 35 d after KA injection. (scale bars = 100um. n=4). All data are shown as the mean ± SEM. *p < 0.05, **p < 0.01. TLE, temporal lobe epilepsy.


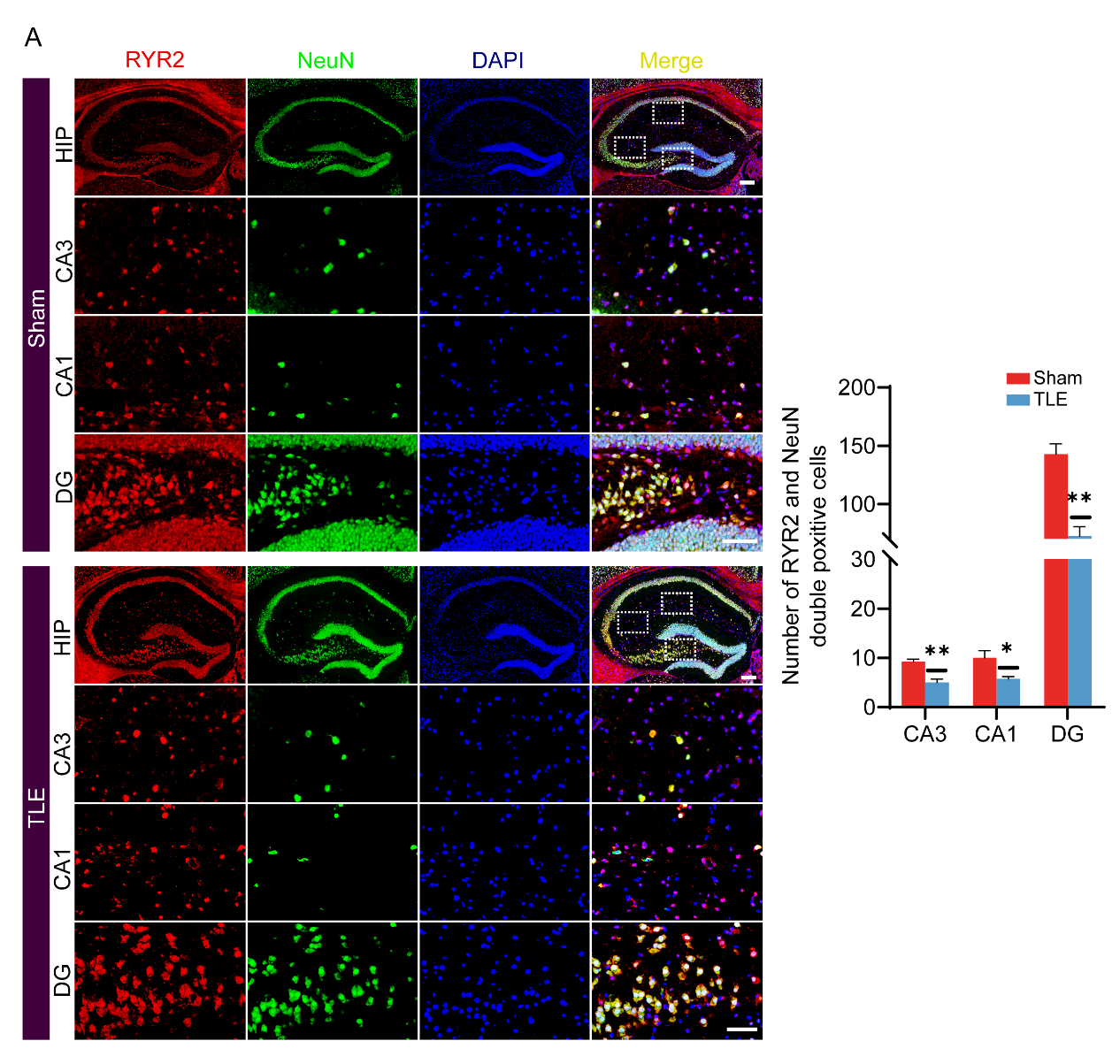


**Figure S9 Expression and activity of RYR2 in hippocampal neural of epileptic mice.** (A) Dual immunofluorescence analysis with anti-RYR2 (red) and anti-NeuN antibodies (green) in hippocampal neural of sham and epileptic mice at 35 d after KA injection. (scale bars = 100um. n=4). All data are shown as the mean ± SEM. ns, not significant. *p < 0.05, **p < 0.01. TLE, temporal lobe epilepsy. HIP, hippocampus.


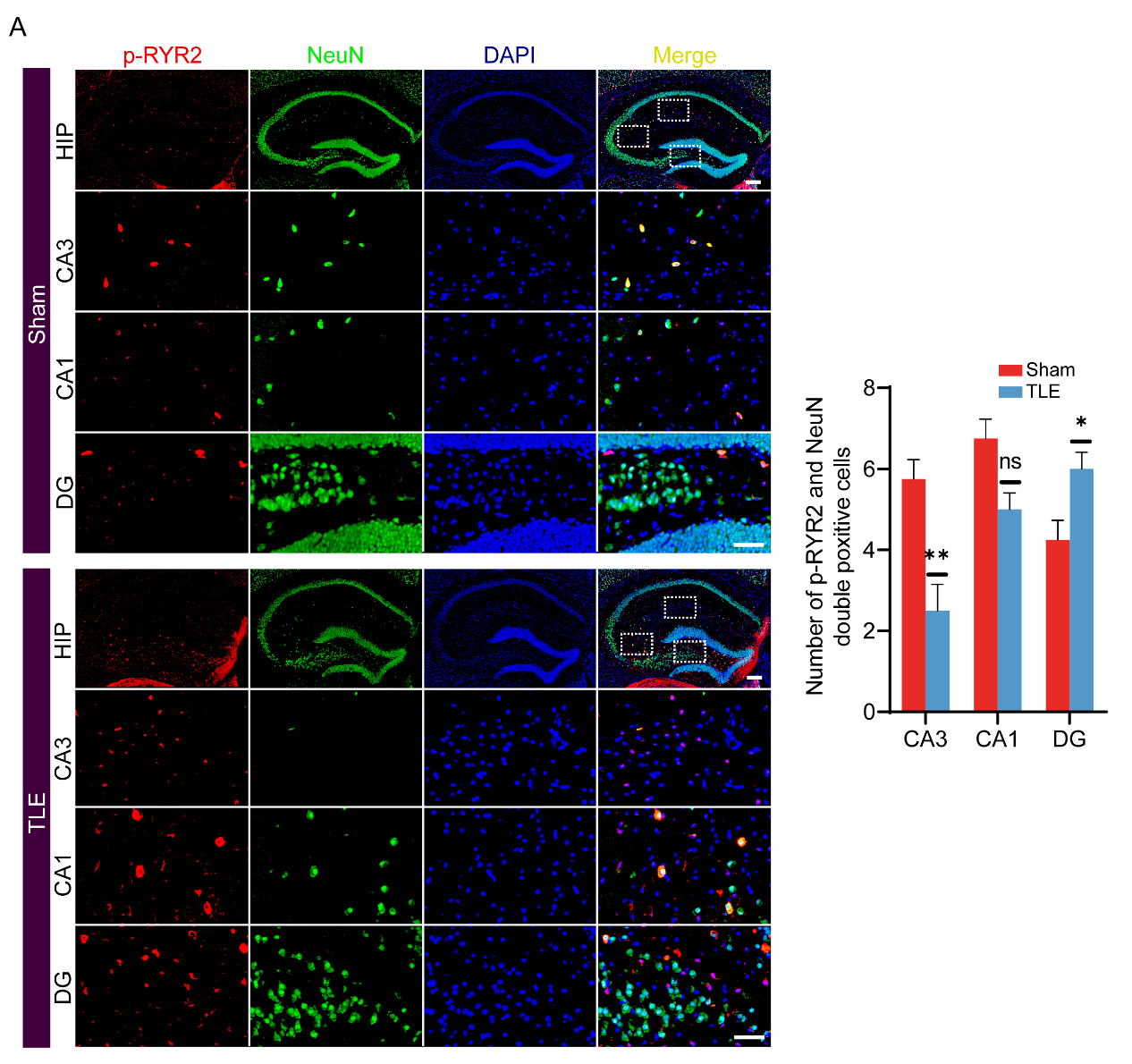


**Figure S10 Expression and activity of p-RYR2 in hippocampal neural of epileptic mice.** (A) Dual immunofluorescence analysis with anti-p-RYR2 (red) and anti-NeuN antibodies (green) in hippocampal neural of sham and epileptic mice at 35 d after KA injection. (scale bars = 100um. n=4). All data are shown as the mean ± SEM. ns, not significant. *p < 0.05, **p < 0.01. TLE, temporal lobe epilepsy. HIP, hippocampus.
